# Neonatal sepsis and mortality in low-income and middle-income countries from a facility-based birth cohort: an international multisite prospective observational study

**DOI:** 10.1016/S2214-109X(22)00043-2

**Published:** 2022-04-12

**Authors:** Rebecca Milton, David Gillespie, Calie Dyer, Khadijeh Taiyari, Maria J Carvalho, Kathryn Thomson, Kirsty Sands, Edward A R Portal, Kerenza Hood, Ana Ferreira, Thomas Hender, Nigel Kirby, Jordan Mathias, Maria Nieto, William J Watkins, Delayehu Bekele, Mahlet Abayneh, Semaria Solomon, Sulagna Basu, Ranjan K Nandy, Bijan Saha, Kenneth Iregbu, Fatima Z Modibbo, Stella Uwaezuoke, Rabaab Zahra, Haider Shirazi, Syed U Najeeb, Jean-Baptiste Mazarati, Aniceth Rucogoza, Lucie Gaju, Shaheen Mehtar, Andre N H Bulabula, Andrew C Whitelaw, Timothy R Walsh, BARNARDS Group, Oludare Odumade, Rozina Ambachew, Zenebe Gebre Yohannes, Gesit Metaferia, Redeat Workneh, Tefera Biteye, Yahya Zekaria Mohammed, Alula M Teklu, Balkachew Nigatu, Wendimagegn Gezahegn, Partha Sarathi Chakravorty, Sharmi Naha, Anuradha Mukherjee, Khairiyya Muhammad Umar, Asunugwo Vivian Akunna, Queen Nsude, Ifeoma Uke, Mary-Joe Okenu, Chinenye Akpulu, Chukwuemeka Mmadueke, Samuel Yakubu, Lamidi Audu, Nura Idris, Safiya Gambo, Jamila Ibrahim, Edwin Chinago, Ashiru Yusuf, Shamsudden Gwadabe, Adeola Adeleye, Muhammad Aliyu, Amina Muhammad, Aishatu Kassim, Aisha Sani Mukaddas, Rashida Yakubu Khalid, Fatima Ibrahim Alkali, Maryam Yahaya Muhammad, Fatima Muhammad Tukur, Surayya Mustapha Muhammad, Adeola Shittu, Murjanatu Bello, Fatima Habib Sa ad, Shaheed Zulfiqar, Adil Muhammad, Muhammad Hilal Jan, Lauren Paterson, Grace J Chan

**Affiliations:** a Institute of Infection and Immunity, Cardiff University, Cardiff, UK; b Centre for Trials Research, Cardiff University, Cardiff, UK; c Institute of Biomedicine, Department of Medical Sciences, University of Aveiro, Aveiro, Portugal; d Ineos Institute of Antimicrobial Research, Department of Zoology, University of Oxford, Oxford, UK; e St Paul's Hospital Millennium Medical College, Addis Ababa, Ethiopia; f Division of Bacteriology, ICMR-National Institute of Cholera and Enteric Diseases Beliaghata, Kolkata, India; g Department of Neonatology, Institute of Postgraduate Medical Education and Research, Kolkata, India; h National Hospital, Abuja, Nigeria; i Murtala Mohammed Specialist Hospital, Kano, Nigeria; j Department of Microbiology, Quaid-i-Azam University, Islamabad, Pakistan; k Pakistan Institute of Medical Sciences, Islamabad, Pakistan; l University Teaching Hospital, Kigali, Rwanda; m Department of Global Health, Stellenbosch University, Cape Town, South Africa; n Infection Control Africa Network, Cape Town, South Africa; o Division of Medical Microbiology at the National Health Laboratory Services Tygerberg and Stellenbosch University, Cape Town, South Africa; p Department of Pediatrics, Boston Children's Hospital, Harvard Medical School, Boston, MA, USA; q Department of Epidemiology, Harvard T H Chan School of Public Health, Boston, MA, USA

## Abstract

**Background:**

Neonatal sepsis is a primary cause of neonatal mortality and is an urgent global health concern, especially within low-income and middle-income countries (LMICs), where 99% of global neonatal mortality occurs. The aims of this study were to determine the incidence and associations with neonatal sepsis and all-cause mortality in facility-born neonates in LMICs.

**Methods:**

The Burden of Antibiotic Resistance in Neonates from Developing Societies (BARNARDS) study recruited mothers and their neonates into a prospective observational cohort study across 12 clinical sites from Bangladesh, Ethiopia, India, Pakistan, Nigeria, Rwanda, and South Africa. Data for sepsis-associated factors in the four domains of health care, maternal, birth and neonatal, and living environment were collected for all mothers and neonates enrolled. Primary outcomes were clinically suspected sepsis, laboratory-confirmed sepsis, and all-cause mortality in neonates during the first 60 days of life. Incidence proportion of livebirths for clinically suspected sepsis and laboratory-confirmed sepsis and incidence rate per 1000 neonate-days for all-cause mortality were calculated. Modified Poisson regression was used to investigate factors associated with neonatal sepsis and parametric survival models for factors associated with all-cause mortality.

**Findings:**

Between Nov 12, 2015 and Feb 1, 2018, 29 483 mothers and 30 557 neonates were enrolled. The incidence of clinically suspected sepsis was 166·0 (95% CI 97·69–234·24) per 1000 livebirths, laboratory-confirmed sepsis was 46·9 (19·04–74·79) per 1000 livebirths, and all-cause mortality was 0·83 (0·37–2·00) per 1000 neonate-days. Maternal hypertension, previous maternal hospitalisation within 12 months, average or higher monthly household income, ward size (>11 beds), ward type (neonatal), living in a rural environment, preterm birth, perinatal asphyxia, and multiple births were associated with an increased risk of clinically suspected sepsis, laboratory-confirmed sepsis, and all-cause mortality. The majority (881 [72·5%] of 1215) of laboratory-confirmed sepsis cases occurred within the first 3 days of life.

**Interpretation:**

Findings from this study highlight the substantial proportion of neonates who develop neonatal sepsis, and the high mortality rates among neonates with sepsis in LMICs. More efficient and effective identification of neonatal sepsis is needed to target interventions to reduce its incidence and subsequent mortality in LMICs.

**Funding:**

Bill & Melinda Gates Foundation.

## Introduction

Neonatal sepsis is a primary cause of neonatal mortality within low-income and middle-income countries (LMICs), with LMICs bearing the burden of 99% of global neonatal mortality, highlighting global disparity.^1^, ^2^ Without significant reduction of infection-related neonatal deaths in LMICs it is unlikely that Sustainable Development Goal 3, which aims to reduce neonatal mortality to at least 12 per 1000 livebirths by 2030 will be met.^3^

Population-level estimates of laboratory-confirmed sepsis in high-income countries are well studied and documented; however, accurate incidence and associations remain decidedly understudied and undetermined in LMICs.^4^, ^5^ Existing research has identified low birthweight, preterm birth (<37 weeks’ gestation), premature rupture of membranes (PROM), neonatal sex, intrapartum-related complications such as perinatal asphyxia, low socioeconomic status, poor sanitation, malnutrition, and overcrowding as associated with an increased risk of neonatal sepsis.^6^, ^7^, ^8^, ^9^ In resource-poor settings, efficient and effective diagnosis of sepsis, including identification and antibiotic susceptibility reporting, is challenging.^10^ To date, no studies supported by microbiological data measure incidence of neonatal sepsis and subsequent mortality within a facility-based cohort. The absence of these data jepordises effective intervention development.^11^, ^12^ To further exacerbate the issue, the global rise in multidrug-resistant bacteria, particularly Gram-negative bacteria, compromises antimicrobial stewardship and increases neonatal mortality and morbidity.^5^, ^13^, ^14^

**Research in context**

**Evidence before this study**

We searched PubMed and Science Direct for articles published in English between Jan 1, 2008, and Dec 31, 2021, using the keywords “neonatal sepsis”, “low- and middle-income countries”, “developing countries”, “Africa”, and “Asia”. Our search strategy yielded 1428 articles of which 72 were relevant. After further scrutiny and excluding reviews, meta-analyses, clinical trials, and focusing on specific cohorts (eg, preterm neonates or those with fungal sepsis) a total of 14 were included. WHO and Save the Children US webpages were consulted to seek additional information. Included articles were examined for richness of epidemiological data, type of institution that conducted the research (public or private institution), single or multisite, definition of sepsis (clinically suspected or laboratory confirmed), and study design. Previous studies on neonatal sepsis focused exclusively on neonatal units within hospitals or are single-site studies. No studies enrolled from a facility-based birth cohort with a denominator of all births. Most studies (12 [92%] of 13) enrolled participants with clinical signs of sepsis from a neonatal intensive care unit and 85% (11 of 13) included laboratory confirmation of sepsis. NeoAMR, ANISA, and the DeNIS study are large neonatal sepsis studies that examined antimicrobial resistance. Both DeNIS and NeoAMR recruited from neonatal intensive care units, and ANISA recruited from the community. NeoOBS study has a similar study design to BARNARDS; however, it is looking at sepsis cases only.

**Added value of this study**

BARNARDS is the first prospective observational study looking at the incidence and associations of neonatal sepsis from a facility-based birth cohort in low-income and middle-income countries (LMICs) supported by positive blood cultures with an aim to reduce the gap in research and data in these areas of high burden. BARNARDS assessed data from four domains, health care, maternal, birth and neonatal, and living environment and explored associations with neonatal sepsis and all-cause mortality. BARNARDS enrolled mothers and neonates from 12 facilities in seven LMICs and excluded wholly private health-care institutions, with the intention of capturing areas of low socioeconomic status often precluded in previous studies. Some of the clinical sites had not participated in many research activities, all of which were representative of usual clinical settings reflective of their region. This study provided a characterisation of the burden of neonatal sepsis and mortality in under-researched populations across several LMICs. The longitudinal nature of BARNARDS supported time-to-event analysis with measures of time to clinically suspected sepsis; time to laboratory-confirmed sepsis; and time to all-cause mortality in neonates with no clinically suspected sepsis and no laboratory-confirmed sepsis, clinically suspected sepsis but no laboratory-confirmed sepsis, and clinically suspected sepsis with laboratory-confirmed sepsis (a sub-set of clinically suspected sepsis).

**Implications of all the available evidence**

Neonatal sepsis and its associated mortality are key global health concerns that are challenging to manage, especially in LMICs. The international network of BARNARDS provides a synergistic blend of clinical, epidemiological, and microbiological data, generating valuable evidence of neonatal sepsis and mortality following sepsis in LMICs.

The Burden of Antibiotic Resistance in Neonates from Developing Societies (BARNARDS) study was established in 2015 to determine the incidence of and factors associated with neonatal clinically suspected sepsis, laboratory-confirmed sepsis, and all-cause mortality across 12 sites in seven LMICs (Bangladesh, Ethiopia, India, Pakistan, Nigeria, Rwanda, and South Africa; appendix p 2). There were two recruitment pathways: a facility-based birth cohort and neonates admitted to hospital with signs of sepsis. This Article focuses on the facility-based birth cohort and aims to provide an understanding of the extent to which neonatal sepsis and subsequent mortality occur across several LMICs in Africa and south Asia. It also aims to identify the factors associated with both neonatal sepsis and all-cause mortality.

## Methods

### Study design and participants

BARNARDS was a large multisite international prospective observational study, recruiting mothers and neonates aged 0–60 days from 12 clinical sites at large public hospitals located in Rwanda, Bangladesh, Ethiopia, Nigeria, Pakistan, India, and South Africa (appendix p 2) between Nov 12, 2015 and Feb 1, 2018. Some sites included community hospitals, providing urban and rural comparisons (appendix p 3). Ethical approval was obtained at each clinical site (appendix p 4). Mothers were provided with study information in local languages. Informed consent (written, if possible, if not, oral) was collected from the mothers by trained researchers. Mothers presenting to BARNARDS’ clinical sites in labour and their respective liveborn neonates were eligible to be enrolled. Stillborn neonates and their respective mothers were ineligible and excluded if inadvertently included.

### Procedures

Demographic and clinical data were collected by standardised electronic or paper-based questionnaires if the internet connection posed issues, or staff safety was compromised (appendix pp 5–8). The questionnaires facilitated the analysis of factors associated with neonatal sepsis and all-cause mortality within four domains: health care, maternal, birth and neonatal, and living environment (panel). Telephone or home-visit follow-ups were conducted at days 3, 7, 14, 28, and 60 of life of neonates by research nurses. Neonates were followed up until 60-days old, loss to follow-up, or death (appendix p 9). All sites were provided with a standardised list of symptoms indicative of neonatal sepsis (appendix p 9). Microbiology methods are published elsewhere.^15^

Panel **Description of health-care domain, variables, and categories analysed in BARNARDS**

**Health-care domain (researcher reported)**

• Type of ward
  • Maternity
  • Neonatal
  • Obstetrics and gynaecology
  • Other
• Number of beds on the ward
  • 1–3
  • 4–10
  • 11–20
  • 21 or more
• Bathroom on the ward
• Mother's location on the ward
  • First third (closest to door)
  • Middle third
  • Last third (furthest from door)
  • Other

**Maternal domain (mother reported)**

• Mother's age
  • 20 years or younger
  • 21–35 years
  • 36 years or older
• Pregnancy history
  • First pregnancy
  • Previous pregnancy did not result in a livebirth
  • Live previous birth
• Health conditions
  • Diabetes
  • Hypertension
  • Immune-compromised
  • Malaria
  • Tuberculosis
  • Other infection
  • Typhoid
  • Other health condition
• Received private health care in previous 3 months
• Visited traditional healer in previous 3 months
• Hospitalised in previous 12 months
• Used antibiotics in previous 3 months
• Mother's educational status
  • None or primary
  • Secondary schooling or university

**Birth and neonatal domain**

• Gestational age, mother-reported
  • Term (37–41 weeks)
  • Preterm (<37 weeks)
  • Post-term (>41 weeks)
• Premature rupture of membranes, mother reported or researcher reported
• Delivery type, researcher reported
  • Natural (spontaneous vaginal delivery)
  • Planned caesarean section
  • Emergency caesarean section
  • Other assisted birth
• Breech, researcher-reported
• Perinatal asphyxia reported, researcher-reported
  • Yes
  • No
  • Unknown
• Multiple birth, mother-reported or researcher-reported

**Living environment domain (mother-reported)**

• Member of household travelled outside city, province, or country in previous 12 months
• Monthly household income (based on local area average)
  • Household income is lower than average
  • Household income is equal to or higher than average
• Type of area
  • Rural
  • Urban
• Semi-rural
• Type of house
  • Apartment
  • Separate house
  • Shack
  • Other
• Number of bedrooms in residence
  • 0
  • 1–2
  • 3 or more
• Number of people residing in the house
  • 1–3
  • 4–6
  • 7 or more
• Primary source of drinking water
  • Municipal network
  • Private well
  • Communal taps
  • Water vendor, sachet, or bottled water
  • Ground water
• Is the water treated
  • Boiled
  • Filtered
  • Neither
• Presence of stagnant or sewage water near home
• Electricity supply in household (based on reported hours or days of availability)
  • No supply
  • Poor supply
  • Intermittent supply
  • Regular supply
• Frequency of solid waste collection, if nearby
  • No solid waste pipe nearby, or other
  • We deal with it ourselves
  • Once a week or more
  • Less than once a week
• Type of toilet
  • Pit latrine or no toilet
  • Sit or squat toilet with flush
• Whether house is served by wastewater network
• Access to soap (of mother)
  • Yes
  • No
  • Sometimes
• Hand washing frequency (of mother)
  • Occasionally
  • Frequently
• Bath or shower frequency (of mother)
  • Occasionally
  • Frequently

### Outcomes

The three primary outcomes were: clinically suspected sepsis, laboratory-confirmed sepsis, and all-cause mortality during the first 60 days of life. Clinically suspected sepsis was defined as the collection of 0·5 mL or more of blood for culture from a neonate where the clinician had identified sepsis symptoms. In neonates with clinically suspected sepsis, laboratory-confirmed sepsis was defined as a positive blood culture. Blood cultures with suspected contaminants (appendix p 10) were excluded from laboratory-confirmed sepsis. All-cause mortality was defined as being reported deceased by a clinician or family member at one of the predefined follow-up points; date of death was recorded to determine neonatal age.

### Statistical analysis

BARNARDS’ countries were chosen to provide an even continental distribution between south Asia and Africa. Numerous factors including economic and health indices, populations, reported incidences of antimicrobial resistance, and neonatal sepsis, as well as veracity of data collection were considered in site selection (appendix p 3). Each site was asked to recruit all eligible mothers over a period of at least 12 months, determined by the duration of funding, and a formal sample size was not calculated.

We estimated the incidence proportion of clinically suspected sepsis and laboratory-confirmed sepsis as the number of cases divided by the number of livebirths, multiplying by 1000 to obtain estimates per 1000 livebirths, and calculating 95% CIs with overall incidence proportions inflated for clustering of neonates within clinical sites using mixed-effects models. As clinically suspected sepsis and laboratory-confirmed sepsis outcomes were based on blood samples being taken at hospital sites, for this outcome no neonates were lost to follow-up and we could estimate incidence proportions.

The incidence rate of all-cause mortality was calculated as the number of confirmed deaths divided by the neonate-time at risk (ie, death, 60 days of life, or final observed timepoint as a censored observation). This was multiplied by 1000 to provide a rate per 1000 neonate-days and accompanied by 95% CIs. We focused on neonate-time as our denominator since not all neonates were followed up for 60 days. The all-cause mortality rate is reported overall, by clinical site, and by sepsis status (no clinically suspected sepsis and no laboratory-confirmed sepsis, clinically suspected sepsis but no laboratory-confirmed sepsis, and clinically suspected sepsis with laboratory-confirmed sepsis [a sub-set of clinically suspected sepsis]). Overall and sepsis status-specific confidence intervals for all-cause mortality rates were inflated for clustering of neonates within clinical sites by calculating jackknife CIs.

We report time from birth until clinically suspected sepsis and laboratory-confirmed sepsis as incidence graphs (appendix p 48), time from birth until all-cause mortality as Kaplan-Meier survival curves, and the ten most common bacterial organisms associated with laboratory-confirmed sepsis as stacked bar charts (appendix p 49).

We fitted bivariable and multivariable regression models to investigate the associations between factors in the health-care, maternal, birth and neonatal, and living-environment domains and clinically suspected sepsis, laboratory-confirmed sepsis, and all-cause mortality. For clinically suspected sepsis and laboratory-confirmed sepsis, we fitted Poisson regression models with robust standard errors and controlling for site as a fixed effect.^16^ Findings are reported as relative risks (RRs) and 95% CIs. For all-cause mortality, we fitted flexible parametric survival models, adjusting for site and sepsis status, with the baseline hazard function modelled as a restricted cubic spline with three knots.^17^ Findings are reported as hazard ratios (HRs) and 95% CIs. For laboratory-confirmed sepsis, we fitted multivariable models adjusting for known and measured confounders based on the minimally sufficient adjustment sets implied by our directed acyclic graphs. Our analytical directed acyclic graphs (appendix pp 11–14) were informed by a review of the literature and expert opinion within the study team. We extended our Poisson regression models, with each model focusing on a single exposure of interest (preterm birth, premature breaking of waters, delivery type, and perinatal asphyxia), and adjustments made for known and measured confounders implied by the directed acyclic graphs. For the premature breaking of waters analysis, we further extended this by including a gestational age × premature breaking of waters interaction to explore whether the adjusted association between premature breaking of waters and laboratory-confirmed sepsis differed depending on gestational age. For all-cause mortality, we explored the association between sepsis status and mortality after adjusting for birth and neonatal factors and markers of socioeconomic status (including type of residence, type of toilet in home, primary source of drinking water, monthly overall household income, and electricity supply in home). All models were fitted within a multiple imputation framework (fully conditional specification with 50 imputed datasets and augmented regression used to handle perfect prediction of categorical variables).^18^ We used site, neonatal sepsis variables, and maternal age to impute missing data as we believed missing data were missing at random given observed data and these were the only complete variables that we believed would be related to the underlying missing mechanism (appendix pp 15–16). Our analysis was not adjusted for clustering of multiple births due to small numbers.^19^, ^20^ All statistical analyses were conducted using Stata (v16.1).

### Role of the funding source

The funder had a minor role in the study design, including site selection and definition of primary outcome. The funder had no role in data analysis, or writing of the manuscript.

## Results

**Figure 1:**
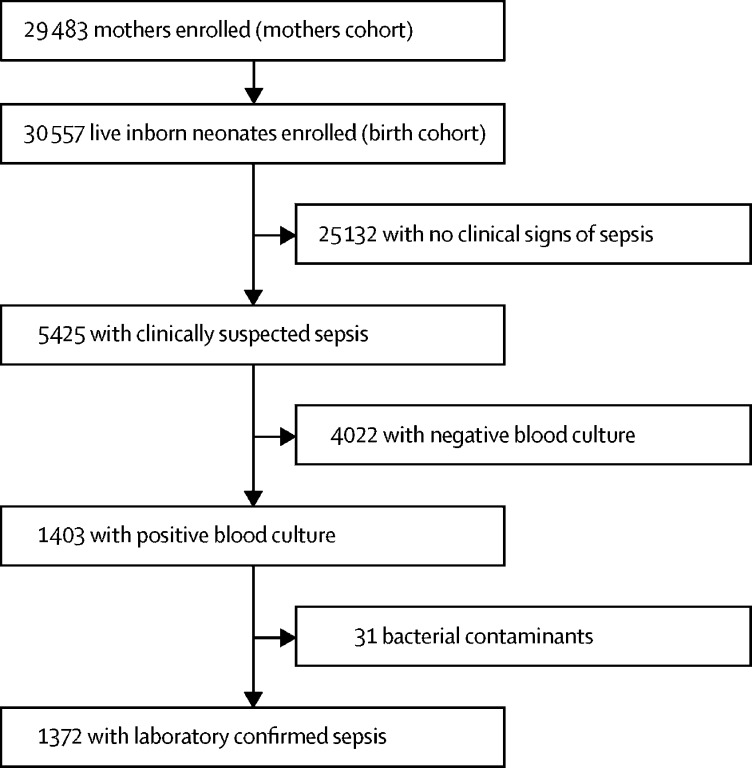
Study profile

BARNARDS enrolled 29 483 mothers and 30 557 neonates (figure 1). Recruitment took place between Nov 12, 2015, and Feb 1, 2018. Most mothers (22 678 [76·9%] of 29 483) were aged between 21 and 35 years; 33·5% (9879 of 29 483) were primigravida, 83·5% (24 621 of 29 483) reported a monthly household income lower than local average, and 66·4% (19 505 of 29 373) were educated to secondary-school level or higher (appendix pp 17–29).

The most frequently reported sources of drinking water included municipal network (8703 [29·7%] of 29 341), private well (7883 [26·9%] of 29 341), and communal taps (6277 [21·4%] of 29 341). Most mothers (28 845 [97·9%] of 29 469) had access to soap; 59·6% reported occasional handwashing (17 563 of 29 701) and 40·1% (11 908 of 29 701) reported frequent handwashing.

Caesarean sections were performed for 11 192 (36·7%) of 30 498 deliveries, with 8055 (72·0%) recorded as emergencies, 3084 (27·5%) reported as planned, and the remainder unknown (<1%). 1784 (42·4%) of 4205 preterm neonates, 877 (53·8%) of 1630 breech presentation neonates, 1055 (43·24%) of 2440 neonates with reported perinatal asphyxia, and 1151 (31·0%) of 3713 neonates born following PROM were all delivered by emergency caesarean section. Preterm neonates (<37 weeks) accounted for 4227 (14·6%) of 29 017 deliveries, and 1568 (5·4%) were post-term (>41 weeks). Additionally, 3715 (12·4%) of 29 936 neonates were delivered following PROM and 2440 (8·0%) of 30 550 had reported perinatal asphyxia.

Of the 30 577 neonates enrolled, 5425 neonates presented with clinical signs of sepsis, with 1372 of these identified as laboratory-confirmed sepsis (figure 1). The overall incidence proportion of clinically suspected sepsis was 166·0 per 1000 livebirths (95% CI 97·69–234·24) and for laboratory-confirmed sepsis, 46·9 per 1000 livebirths (19·04–74·79; table 1).

**Table 1:** Incidence proportions of clinically suspected and laboratory-confirmed sepsis presented by site and overall

	**Setting**	**Neonates enrolled**	**Neonates with CSS**	Incidence proportion of CSS (95% CI)*	**Neonates with CSS and LCS**	Incidence proportion of CSS and LCS (95% CI)*
Bangladesh, Chittagong, Chattogram Maa-O-Shishu Hospital Medical College	Urban	563	126	223·8 (189·4–258·2)	37	65·7 (45·3–86·2)
Bangladesh, Kumudini, Kumudini Hospital	Rural	1386	35	25·3 (17·0–33·5)	4	2·9 (0·1–5·7)
Ethiopia, Addis Ababa, St Paul's Hospital Millennium Medical, College	Urban	4187	479	114·4 (104·8–124·0)	183	43·7 (37·5–49·9)
India, Kolkata, National Institute of Cholera and Enteric Diseases	Urban	1126	44	39·1 (27·8–50·4)	8	7·1 (2·2–12·0)
Nigeria, Kano, Murtala Muhammad Specialist Hospital	Rural	5584	531	95·1 (87·4–102· 8)	94	16·8 (13·5–20·2)
Nigeria, Abuja, National Hospital Abuja	Urban	1531	303	197·9 (178·0–217·9)	101	66·0 (53·5–78·4)
Nigeria, Abuja, Wuse District Hospital	Urban	2224	102	45·9 (37·2–54·6)	31	13·9 (9·1–18·8)
Pakistan, Bhara Kahu, Community site	Rural	415	101	243·4 (202·1–284·7)	34	81·9 (55·5–108·3)
Pakistan, Islamabad, Pakistan Institute of Medical Sciences	Urban	7197	2364	328·5 (317·6–339·3)	604	83·9 (77·5–90·3)
Rwanda, Kigali, The University Teaching Hospital of Kigali	Urban	1173	299	254·9 (230·0–279·8)	50	42·6 (31·1–54·2)
Rwanda, Kabgayi, Kabgayi Hospital	Rural	2005	481	239·9 (221·2–258·6)	149	74·3 (62·8–85·8)
South Africa, Cape Town, Tygerberg Hospital	Urban	3166	560	176·9 (163·6–190·2)	77	24·3 (19·0–29·7)
Total	..	30 557	5425	166·0 (97·7–234·2)	1372	46·9 (19·0–74·8)

Data are n or incidence proportion (95% CI). Overall estimates are inflated for clustering of neonates within sites. CSS=clinically suspected sepsis. LCS=laboratory-confirmed sepsis.

* Estimates are reported per 1000 livebirths.

There were 877 neonatal deaths recorded within the first 60 days of life over 1 055 284 neonate-days at risk. Full follow-up data (ie, alive at or deceased by the end of the study) were available for 16 789 (54·9%) of 30 557 neonates, with the remainder censored at their final observation point (median 7 days, IQR 1–56 days). The overall incidence rate of all-cause mortality was 0·83 per 1000 neonate-days (95% CI 0·37–2·00). In neonates with no clinically suspected sepsis and no laboratory-confirmed sepsis, the incidence rate of all-cause mortality was 0·40 per 1000 neonate-days (0·25–0·62). Those with clinically suspected sepsis but no laboratory-confirmed sepsis had an all-cause mortality incidence rate of 2·86 per 1000 neonate-days (1·34–7·60) and for those with clinically suspected sepsis and laboratory-confirmed sepsis it was 5·65 per 1000 neonate-days (3·00–13·35; appendix p 30). The incidence rates of clinically suspected sepsis, laboratory-confirmed sepsis, and all-cause mortality varied across the clinical sites (Table 1, Table 2).

**Table 2:** Numbers of neonates deceased by sepsis status, site, and overall

	**Setting**	**Neonates enrolled (n)**	**Neonates without CSS or LCS deceased (n)**	Incidence of mortality per 1000 neonate-days in neonates without CSS or LCS (95%CI)*	**Neonates with CSS without LCS deceased (n)**	Incidence of mortality per 1000 neonate-days in neonates with CSS without LCS (95%CI)*	**Neonates with CSS and LCS deceased (n)**	Incidence of mortality per 1000 neonate-days in neonates with CSS and LCS (95%CI)*
Bangladesh, Chittagong, Chattogram Maa-O-Shishu Hospital Medical College	Urban	563	16	0·86 (0·49–1·40)	2	0·43 (0·05–1·54)	4	2·36 (0·64–6·04)
Bangladesh, Kumudini, Kumudini Hospital	Rural	1386	29	0·69 (0·46–0·99)	3	4·85 (1·00–14·16)	1	15·63 (0·40–87·06)
Ethiopia, Addis Ababa, St Paul's Hospital Millennium Medical College	Urban	4187	53	0·42 (0·32–0·55)	25	2·92 (1·89–4·30)	24	4·40 (2·82–6·55)
India, Kolkata, National Institute of Cholera and Enteric Diseases	Urban	1126	17	0·93 (0·54–1·48)	3	3·18 (0·66–9·29)	2	5·80 (0·70–20·94)
Nigeria, Kano, Murtala Muhammad Specialist Hospital	Rural	5584	65	0·24 (0·19–0·31)	17	0·77 (0·45–1·24)	4	0·84 (0·23–2·16)
Nigeria, Abuja, National Hospital Abuja	Urban	1531	20	0·32 (0·20–0·50)	38	7·30 (5·17–10·03)	16	5·75 (3·29–9·34)
Nigeria, Abuja, Wuse District Hospital	Urban	2224	25	0·24 (0·15–0·35)	6	1·98 (0·73–4·32)	3	2·27 (0·47–6·62)
Pakistan, Bhara Kahu, Community site	Rural	415	7	0·83 (0·34–1·72)	8	7·01 (3·02–13·80)	11	18·12 (9·05–32·43)
Pakistan, Islamabad, Pakistan Institute of Medical Sciences	Urban	7197	83	0·87 (0·69–1·08)	173	4·91 (4·21–5·70)	92	10·03 (8·09–12·30)
Rwanda, Kigali, The University Teaching Hospital of Kigali	Urban	1173	10	4·10 (1·96–7·53)	26	5·83 (3·81–8·54)	5	5·54 (1·80–12·92)
Rwanda, Kabgayi, Kabgayi Hospital	Rural	2005	3	0·25 (0·05–0·73)	10	3·21 (1·54–5·91)	6	4·33 (1·59–9·43)
South Africa, Cape Town, Tygerberg Hospital	Urban	3166	35	0·23 (0·16–0·33)	20	0·75 (0·46–1·16)	15	3·83 (2·14–6·32)
Total	..	30 557	363	0·40 (0·25–0·62)	331	2·86 (1·34–7·60)	183	5·65 (3·00–13·35)

Data are n or incidence proportion (95% CI). CSS=clinically suspected sepsis. LCS=laboratory-confirmed sepsis.

* CI values are inflated for clustering of neonates within sites.

Blood culture results from 31 neonates with clinically suspected sepsis contained suspected bacterial contaminants and did not meet our laboratory-confirmed sepsis definition (appendix p 10). Of the 31 neonates, five were reported deceased.

Associations with clinically suspected sepsis are fully detailed in appendix pp 31–36. Some factors found to be associated with a higher risk of clinically suspected sepsis included private health care within 3 months before enrolment, living in a separate house compared with an apartment, having no bedrooms in the house compared with one or more rooms, drinking water from a private well, communal taps, or groundwater compared with municipal network and living in a rural environment compared with an urban environment.

Health-care factors associated with a higher risk of laboratory-confirmed sepsis included being on a neonatal ward compared with a maternity ward (RR 7·73, 95% CI 6·31–9·46), having a bathroom on the ward compared with no bathroom (1·78, 1·28–2·46), and being situated at the entrance to or in the middle of the ward compared with furthest from the door (1·42, 1·21–1·67 for entrance and 1·42, 1·20–1·67 for middle; appendix pp 37–40).

Maternal factors associated with a higher risk of laboratory-confirmed sepsis included maternal use of antibiotics within 3 months before enrolment (RR 1·48, 95% CI 1·22–1·82) compared with no antibiotic use, maternal hypertension compared with no hypertension (1·73, 1·47–2·04), and being hospitalised in the preceding 12 months compared with no hospitalisation (1·53, 1·27–1·85; appendix pp 37–40).

Birth and neonatal factors associated with a higher risk of laboratory-confirmed sepsis included preterm delivery (RR 3·94, 95% CI 3·53–4·39), PROM (1·64, 1·44–1·87), caesarean section delivery compared with spontaneous vaginal delivery (1·50, 1·23–1·84 for elective and 1·92, 1·69–2·18 for emergency), reported perinatal asphyxia compared with no asphyxia (3·82, 3·38–4·31), being part of multiple births compared with singleton (1·68, 1·40–2·01), and breech delivery compared with spontaneous vaginal delivery (2·02, 1·70–2·41; appendix pp 37–40).

Living-environment factors associated with a higher risk of laboratory-confirmed sepsis included having at least average monthly household income compared with lower than average income (RR 1·22, 95% CI 1·03–1·44), living in a household served by a wastewater network compared with those who were not (1·26, 1·11–1·43), primary source of drinking water being from a water vendor, sachet, or bottled water compared with municipal network (1·31, 1·03–1·65), and living in a rural environment, compared with urban (1·26, 1·11–1·43; appendix pp 37–40).

The multivariable analysis identified that the associations between our exposures of interest (preterm delivery, PROM, delivery type, and reported perinatal asphyxia) and laboratory-confirmed sepsis remained statistically significant after adjusting for known confounding factors (table 3; appendix pp 11–14). There was further evidence to suggest that the association between PROM and laboratory-confirmed sepsis was stronger in term neonates (RR 1·56, 1·28–1·89) compared with preterm (0·99, 0·83–1·19) and post-term neonates (1·27, 0·55–2·93; p-value for gestational age × PROM interaction=0·004; appendix p 41).

**Table 3:** Associations with laboratory-confirmed sepsis unadjusted and adjusted for known confounders

	**Adjusted for site only, relative risk (95% CI)**	Adjusted for site and known factors,*relative risk (95% CI)
**Gestational age**		
Term	(1) ref	(1) ref
Preterm	3·93 (3·53–4·40)	3·64 (3·22–4·10)
Post-term	0·93 (0·68–1·27)	0·91 (0·67–1·25)
**PROM**		
No	(1) ref	(1) ref
Yes	1·64 (1·44–1·87)	1·57 (1·37–1·79)
**Delivery type**		
Spontaneous vaginal delivery	(1) ref	(1) ref
Planned caesarean section	1·50 (1·22–1·84)	1·48 (1·21–1·81)
Emergency caesarean section	1·92 (1·69–2·19)	1·64 (1·44–1·88)
Other assisted birth	0·82 (0·70–0·97)	0·78 (0·66–0·92)
**Perinatal asphyxia**		
No	(1) ref	(1) ref
Yes	3·82 (3·38–4·31)	2·59 (2·25–2·98)
Unknown	2·43 (1·36–4·33)	2·74 (1·24–4·16)

PROM=premature rupture of membranes.

* Known factors informed by minimally sufficient adjustment sets informed by directed acyclic graphs. Known factors in gestational age model were birth as part of a multiple, maternal hypertension, maternal age, maternal infection in the 3 months before enrolment, parity, type of residence, type of toilet in the home, primary source of drinking water, overall household income per month, and electricity supply in the home. Known factors in PROM model were birth as part of a multiple, maternal hypertension, maternal age, maternal infection in the 3 months before enrolment, parity, type of residence, type of toilet in the home, primary source of drinking water, overall household income per month, and electricity supply in the home. Known factors in delivery type model were birth as part of a multiple, perinatal asphyxia, maternal age, maternal infection in the 3 months before enrolment, parity, PROM, type of residence, type of toilet in the home, primary source of drinking water, overall household income per month, and electricity supply in the home. Known factors in perinatal asphyxia model were birth as part of a multiple, maternal hypertension, maternal age, maternal infection in the 3 months before enrolment, parity, and PROM.

Neonates with either clinically suspected sepsis only or clinically suspected sepsis and laboratory-confirmed sepsis had higher rates of all-cause mortality than those without sepsis (HR 5·00, 95% CI 4·26–5·88 for clinically suspected sepsis and 8·95, 7·43–10·79 for laboratory-confirmed sepsis). Neonates situated on a neonatal ward compared with a maternity ward (1·88, 1·38–2·55) were associated with higher rates of all-cause mortality. Maternal hospitalisation in the preceding 12 months compared with no hospitalisation (1·29, 1·02–1·63) and maternal hypertension, compared with no reported hypertension (1·29, 1·05–1·59) were associated with higher rates of neonatal all-cause mortality (appendix pp 42–44).

Preterm delivery was associated with higher rates of all-cause mortality compared with term delivery (HR 4·00, 95% CI 3·41–4·69), as was reported perinatal asphyxia compared with no reported asphyxia (3·74, 3·16–4·42) and multiple births compared with singleton (2·91, 2·42–3·51; appendix pp 42–44).

After adjusting for birth and neonatal factors and markers of socioeconomic status, the all-cause mortality rate remained higher for those with clinically suspected sepsis (HR 2·89, 95% CI 2·42–3·44) and those with clinically suspected sepsis and laboratory-confirmed sepsis (4·39, 3·59–5·37), than compared with neonates with no sepsis within the first 60 days of life.

Living-environment factors associated with higher rates of all-cause mortality included living in a household with a monthly income of at least average compared with lower than average (HR 1·27, 95% CI 1·01–1·60), living in a shack compared with an apartment (1·37, 1·00–1·87), and residing in a rural area compared with urban area (1·20, 1·02–1·41; appendix pp 42–44).

**Figure 2:**
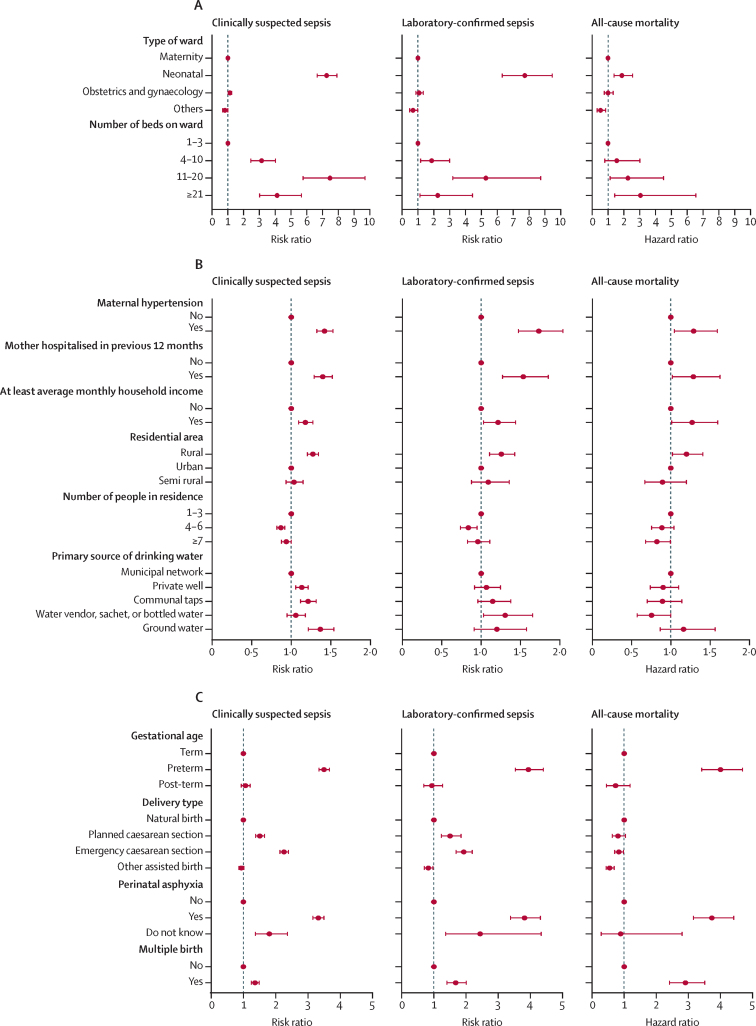
Forest plots of bivariable associations with clinically suspected sepsis, laboratory-confirmed sepsis, and all-cause mortality Forest plot of health-care factors associated with clinically suspected sepsis, laboratory-confirmed sepsis, and all-cause mortality in facility-born neonates in seven LMICs (A). Forest plot of maternal factors and living environment factors associated with clinically suspected sepsis, laboratory-confirmed sepsis, and all-cause mortality among facility-born neonates in seven LMICs (B). Forest plot of birth and neonatal factors associated with clinically suspected sepsis, laboratory-confirmed sepsis, and all-cause mortality among facility-born neonates in seven LMICs (C). Red circles are point estimates and black lines represent 95% CIs. Reference categories are indicated by the presence of a point estimate only. LMICs=low-income and middle-income countries.

Figures 2A–C are forest plots representing all statistically significant findings across clinically suspected sepsis, laboratory-confirmed sepsis, and all-cause mortality. These findings are shown in tabular format in the appendix (pp 45–47).

Time to laboratory-confirmed sepsis by age in days at clinical diagnosis (n=1215) was examined. The greatest proportion of laboratory-confirmed sepsis cases occurred within the first 3 days of life (881 [72·5%] of 1215) and within the first 7 days of life (1078 [88·7%] of 1215; median 1 day, IQR 0–3 days). These results closely aligned with the time from birth until clinically suspected sepsis (median 1 day, IQR 0–2 days; appendix p 48).

**Figure 3:**
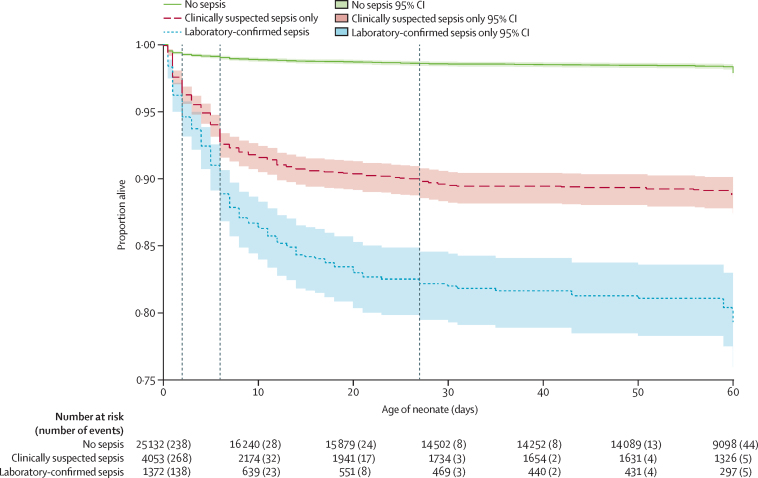
Kaplan-Meier time to all-cause mortality by sepsis status for the first 60 days of life in facility-born neonates in seven low-income and middle-income countries Dashed vertical lines represent days 3, 7, and 28 of life. Y-axis begins at 0·75.

Of the 877 neonates who died, 66% (n=581) died within the first 7 days of life and 88% (n=770) within the first 28 days. Time from birth to all-cause mortality is illustrated in figure 3.

In total, 49 different bacterial species causing sepsis were identified by whole genome sequencing methods.^15^ The five most frequently identified bacteria included *Klebsiella pneumoniae* (n=123), *Klebsiella michiganensis* (n=76), *Staphylococcus aureus* (n=63), *Serratia marcescens* (n=49), and *Burkholderia cenocepacia* (n=44). *Escherichia coli* (n=34)*, Enterobacter cloacae* (n=33), and *Acinetobacter* spp (n=17) were identified in all countries (appendix p 49). Of 123 *K pneumoniae*-associated sepsis cases, 56·9% (n=70) were delivered by caesarean section, and of 34 *E coli*-associated sepsis cases, 58·8% (n=20) were delivered by caesarean section.

## Discussion

BARNARDS assessed the incidence and factors associated with neonatal sepsis and all-cause mortality in seven LMICs representing a range of geographical and economical settings. Compared with neonates with no clinically suspected sepsis and those with no laboratory-confirmed sepsis, all-cause mortality was considerably higher among those with clinically suspected sepsis and even higher again for those with laboratory-confirmed sepsis, confirming that neonatal sepsis is a great contributor to neonatal mortality.

The incidence of clinically suspected sepsis was highly variable across clinical sites potentially due to varying methods of clinical practice and contextual factors—eg, laboratory equipment and population served. Within the DeNIS study, incidence of clinically suspected sepsis was 143 per 1000 admissions; lower than BARNARDS. However, incidence of laboratory-confirmed sepsis was 62 per 1000 livebirths**;** higher than BARNARDS. Inclusion criteria for DeNIS study included admission into a neonatal intensive care unit^14^ whereas BARNARDS included all facility-born neonates. In BARNARDS, the highest incidence rate of all-cause mortality was in neonates with laboratory-confirmed sepsis (5·65 per 1000 neonate-days). Proportionately, the number of deaths recorded in those excluded due to suspected bacterial contaminants was consistent with the all-cause mortality rate in those with laboratory-confirmed sepsis.

Across all four domains we identified associations with increased risks of clinically suspected sepsis, laboratory-confirmed sepsis, and of all-cause mortality. Poor sanitation and living conditions are associated with neonatal sepsis.^9^ We found mothers from households with no household electricity supply had an increased risk of clinically suspected sepsis and laboratory-confirmed sepsis, this could be due to vertical transmission. No evidence of an association between maternal education and neonatal sepsis was found, consistent with existing work.^21^ A counterintuitive finding was the association between clinically suspected sepsis, laboratory-confirmed sepsis, and all-cause mortality and at least average monthly household income, suggesting a higher household income is associated with a poorer neonatal outcome. This finding might reflect reporting biases, accessibility to, and availability of health care or that household income might not be representative of the wealth of the mother.

Increased rates of neonatal sepsis have been reported in mothers with diabetes or hypertension in high-income countries.^22^ In BARNARDS, women with maternal hypertension had an increased risk of delivering a neonate with clinically suspected sepsis, laboratory-confirmed sepsis, and all-cause mortality. PROM is a recognised association with neonatal sepsis, first recorded in 1963;^23^ our findings were consistent with existing literature.^6^ PROM is associated with breech presentation^24^ and we found associations between clinically suspected sepsis, laboratory-confirmed sepsis, and breech presentation, which is unreported elsewhere; a probable cause for this finding is the known connection between PROM and neonatal sepsis. There are known links between maternal malaria and neonatal sepsis;^25^ however, these were not identified in BARNARDS. Location in the hospital after the delivery, such as being on a larger hospital ward (>11 beds), located closest to the door or in the middle of the ward, and being situated on a neonatal ward compared with a maternity ward (this would only occur if the neonate needed treatment, a probable reason for this finding) were also associated with increased risks of neonatal sepsis and all-cause mortality.

Clinical interventions such as prenatal antibiotic use and caesarean sections were associated with neonatal sepsis. Increased risks of clinically suspected sepsis and laboratory-confirmed sepsis associated with prenatal antibiotic use might be related to the disruption of the mother's vaginal microbiota, increasing exposure to sepsis-causing bacteria—eg, *Enterobacterales*. Further research is needed to determine the effect of prenatal antibiotics on neonates’ health; further works could include an in-depth evaluation into antibiotic type, duration, and reason for use to provide a comprehensive understanding of this association. Caesarean sections, elective and emergency, were associated with clinically suspected sepsis and laboratory-confirmed sepsis when compared with spontaneous vaginal delivery. Emergency caesarean sections carried an increased risk, with these neonates likely to have additional risk factors. For example, 1784 (42·4%) of 4205 preterm neonates, 877 (53·8%) of 1630 breech presentation neonates, 1055 (43·2%) of 2440 neonates with reported perinatal asphyxia, and 1151 (31·0%) of 3713 neonates delivered following PROM were delivered by emergency caesarean sections. A study investigating PROM deliveries in Kosovo reported 28% caesarean sections rate.^26^ Neonates delivered via caesarean section are not exposed to the mothers’ vaginal microbiota and might be at increased risk of exposure to pathogenic bacteria, such as staphylococci and *Enterobacterales*.^27^ Performing caesarean sections is a reported risk factor for acquisition of extended-spectrum β-lactamase-producing *Enterobacterales,* bacteria which commonly cause sepsis in health-care settings.^28^ Caesarean sections inevitably lead to longer hospital stays compared with spontaneous vaginal delivery, increasing chances of neonatal sepsis.^7^ This said, our findings indicate an association between emergency caesarean sections and a lower rate of all-cause mortality, potentially because the reason for the caesarean section was unrelated to neonatal sepsis. Higher risks of clinically suspected sepsis and laboratory-confirmed sepsis were identified in women in their first pregnancy compared with those who had previously had a livebirth, congruent with existing research.^7^, ^29^ A plausible explanation for this is longer labour or complications associated with first pregnancies.^30^

The management of neonatal sepsis in LMICs is challenging and increasingly complex due to increased levels of antimicrobial resistance and poor resources for diagnosis and treatment. Similar to other studies, we found that the majority of neonates developed sepsis within the first 24–48 h of life.^14^, ^29^ This finding signals a need for improvement in rapid diagnostic methods and targeted treatment.^31^ A recent review has highlighted the use and importance of multi-omics to improve the accuracy of sepsis biomarkers and the use of health systems data for tailoring digital diagnostics.^32^ Basic interventions such as targeted infection prevention control and implementation of water, sanitation, and hygiene measures (known as WASH),^33^ which contribute to the prevention of spread of antimicrobial resistance, and therefore reduce the incidence of neonatal sepsis.^33^ Gaining an understanding of associations with neonatal sepsis and mortality is helpful for informing interventions to combat and reduce neonatal sepsis in LMICs; through BARNARDS we are able to contribute to this vital evidence base.

BARNARDS consisted of a wide range of clinical sites, representative of usual clinical settings and some non-research sites. Our results provide an accurate characterisation of the burden of neonatal sepsis and mortality in under-served and understudied populations across several LMICs. However, we note two limitations with regard to our data and analysis. First, the definition of laboratory-confirmed sepsis is considered to be fewer than four colony-forming units of bacteria per mL of blood.^34^ Obtaining adequate volume for blood culture samples, particularly from preterm neonates, is challenging. Small volumes of blood reduce the probability of detecting bacterial organisms, which might result in underestimated prevalence of laboratory-confirmed sepsis.^35^ Second, differing interpretations of clinically suspected sepsis were apparent despite standardised operating procedures.^35^ Some laboratory-confirmed sepsis isolates were lost at clinical sites or in transit to the laboratory at Cardiff University (UK). Third, not all neonatal outcomes were recorded (lost to follow-up). Therefore, all-cause mortality might be under-reported as 60-day follow-up was challenging. The decision to analyse time to all-cause mortality, incorporating all data available and applying censoring to neonates lost to follow-up therefore yields a lower bound for these incidence estimates.

The use of purposive sampling to select clinical sites and countries, in addition to a scarcity of screening data, means that caution should be taken when generalising these findings within countries or continents. Causal relations cannot be drawn from observational studies due to residual confounding.

Within BARNARDS, facility-based cohort data and laboratory methods were generated to improve the identification of neonatal sepsis at a scale not previously seen within this population. BARNARDS highlights the burden of neonatal sepsis in LMICs, and identifies a multitude of factors associated with clinically suspected sepsis, laboratory-confirmed sepsis, and all-cause mortality. Several important knowledge gaps remain, including an urgent need for studies looking at simple and sustainable interventions to reduce the burden of neonatal sepsis.

## Data sharing

Data will be made available upon request from researchers who provide a methodologically sound proposal, following assessment from the research team, and subject to a data sharing agreement. Data will be de-identified and will strictly adhere to patient confidentiality and consent. All relevant study protocols including ethical approvals are on the BARNARDS group website (https:/www.ineosoxford.ox.ac.uk/research/barnards). Datasets specific to this study will be made available upon request following publication.

## Declaration of interests

We declare no competing interests.
