# Supplementary material for: Neonatal sepsis and mortality in low-income and middle-income countries from a facility-based birth cohort: an international multisite prospective observational study
Source: Lancet Glob Health. 2022 Apr 12;10(5):e661–72. doi: 10.1016/S2214-109X(22)00043-2 (PMC9023753; doi:10.1016/S2214-109X(22)00043-2)
Supplement: Supplementary appendix [file mmc1.pdf]

THE LANCET
Global Health

Supplementary appendix

This appendix formed part of the original submission and has been peer reviewed. We post it as supplied by the authors.

Supplement to: Milton R, Gillespie D, Dye C, et al. Neonatal sepsis and mortality in low-income and middle-income countries from a facility-based birth cohort: an international multisite prospective observational study. *Lancet Glob Health* 2022; **10:** e661–72.

APPENDIX

Supplementary Table 1a: Literature Review

Search	Neonatal sepsis AND low- and middle-income countries	Neonatal sepsis AND developing countries	Neonatal sepsis AND Africa	Neonatal sepsis AND Asia
Total	205	597	292	334
Number selected for abstract review	19	22	13	18

Supplementary Table 1b: Literature Review

Title	Countries / Sites (n)	NICU enrolment	Sample Size	Lab confirmed	Cases (n)	Public / Private
Clinical features, antimicrobial susceptibility patterns and genomics of bacteria causing neonatal sepsis in a children's hospital in Vietnam: protocol for a prospective observational study. doi: 10.1136/bmjopen-2017-019611	1 / 1	Yes	TBC	Yes	TBC	
Late-onset neonatal sepsis in Arab states in the Gulf region: two-year prospective study. doi: 10.1016/j.ijid.2017.01.006	3 / 5	Yes	67474	Yes	785	Mixture
Culture-proven early-onset neonatal sepsis in Arab states in the Gulf region: two-year prospective study. doi: 10.1016/j.ijid.2016.12.006	3 / 5	Yes	67474	Yes	102	Mixture
Towards understanding global patterns of antimicrobial use and resistance in neonatal sepsis: insights from the NeoAMR network. doi: 10.1136/archdischild-2019-316816	12 / 39	Yes	?	Yes	1635	Mixture
Epidemiology of Neonatal Sepsis and Associated Factors Implicated: Observational Study at Neonatal Intensive Care Unit of Arsi University Teaching and Referral Hospital, South East Ethiopia. doi: 10.4314/ejhs.v29i3.5.	1 / 1	Yes	303	Yes	88	
Neonatal morbidity and mortality in Hargeisa, Somaliland: an observational, hospital based study. doi: 10.11604/pamj.2020.37.3.24741	1 / 1	Yes	164	No	44	
Etiology, Antibiotic Resistance and Risk Factors for Neonatal Sepsis in a Large Referral Center in Zambia. doi: 10.1097/INF.0000000000001154.	1 / 1	Yes	313	Yes	103	
Blood culture result profile and antimicrobial resistance pattern: a report from neonatal intensive care unit (NICU), Asella teaching and referral hospital, Asella, south East Ethiopia. doi: 10.1186/s13756-019-0486-6	1 / 1	Yes	303	Yes	88	
Pattern and etiology of culture-proven early-onset neonatal sepsis: a five-year prospective study. doi: 10.1016/j.ijid.2011.05.004	1 / 1	Yes	56134	Yes	153	Private
Efficacy of an infection control programme in reducing nosocomial bloodstream infections in a Senegalese neonatal unit. doi: 10.1016/j.jhin.2011.04.007	1 / 1	Yes	273	Not all	86	
Characterisation and antimicrobial resistance of sepsis pathogens in neonates born in tertiary care centres in Delhi, India: a cohort study. doi: 10.1016/S2214-109X(16)30148-6	1 / 3	Yes	13530	Yes	1934	
Laboratory-confirmed bloodstream infections in two large neonatal units in sub-Saharan Africa. doi: 10.1016/j.ijid.2020.11.169	1 / 2	Yes	4013	Yes	284	
Causes and incidence of community-acquired serious infections among young children in south Asia (ANISA): an observational cohort study. doi: 10.1016/S0140-6736(18)31127-9	3 / 5	No	63114	Yes	6022	Community
Clinical presentation and 28-day mortality in hospitalized neonates and young infants with clinical sepsis: the global NeoOBS observational cohort (preprint)	11 / 19	Hospitalised	3204	Yes	693	

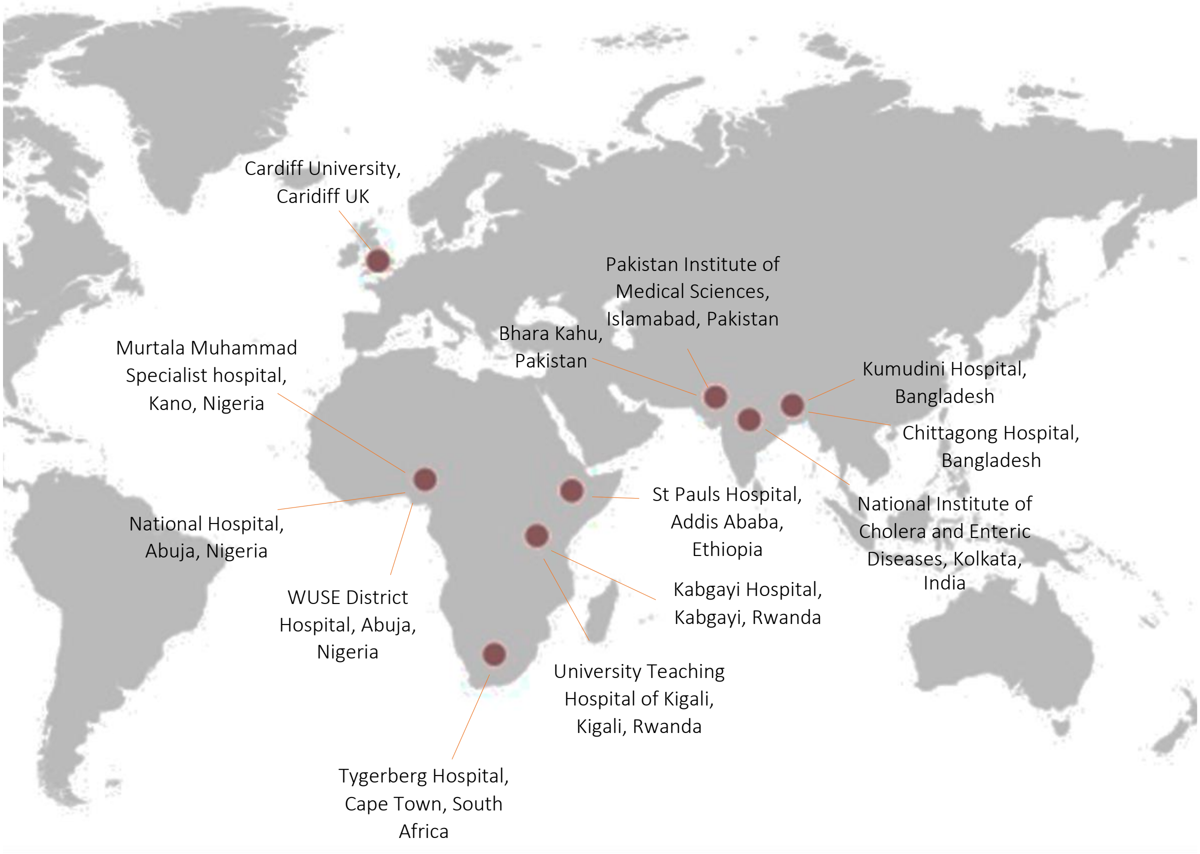

Supplementary Figure. 1. BARNARDS' centres including clinical sites.

Supplementary Table 2: Population, Gross Domestic Product (GDP), Purchasing Power Parity (PPP), Environmental Performance Index (EPI), total health expenditure and reported neonatal mortality reported from countries involved in BARNARDS.

**Country**	Population (millions)*	GDP current US$*	GDP, PPP (current international $)*	EPI (score and country ranking (denominator 180)**	Total health expenditure per captia ($US)*	Neonatal mortality per 1,000 livebirths*
**Bangladesh**	163	325.25 billion	837.064 billion	29 162/180	41.91	19.1
**Ethiopia**	112	107.65 billion	278.552 billion	34.4 134/180	24.23	27.6
**India**	1380	2.63 trillion	8.907 trillion	27.6 168/180	72.87	21.7
**Nigeria**	206	432.29 billion	1.07 trillion	31 151/180	83.74	35.9
**Pakistan**	220	263.68 billion	1.07 trillion	33.1 142/180	42.87	41.2
**Rwanda**	13	10.33 billion	28.68 billion	33.8 137/180	58.31	15.9
**South Africa**	59	301.92 billion	717.38 billion	43.1 95/180	525.96	11.5

*World Bank 2020 (https://databank.worldbank.org) accessed August 2021
** Envirnmental Performance Index (https://epi.yale.edu/epi-results/2020/component/epi) accessed August 2021

Countries were selected to provide a continental distribution between South Asia and Africa. Factors influencing site selection included antibiotic resistance rates, economic and health indices, veracity of data collection, adherence to agreed SOPs, local ethical clearance, and rigorous patient consent.

Supplementary Table 3: **Ethical approval**

**Site**	**Committees**	**Named PI**	**Reference(s)**	**Approval date(s)**
**BC**	Ethical Review Committee, Bangladesh Institute of Child Health	Samir Kumar Saha	BICH-ERC-4/3/2015	15/09/2015
**BK**	Ethical Review Committee, Bangladesh Institute of Child Health	Samir Kumar Saha	BICH-ERC-4/3/2015	15/09/2015
**ES**	Boston Children's Hospital	Grace Chan	IRB-P00023058	11/08/2016
**IN**	Institutional Ethics Committee, National Institute of Cholera and Enteric Diseases and Institue of Post Graduate Medical Education and Research, IPGME&R Research Oversight Committee	Sulagna Basu	A-I/2016-IEC and Inst/IEC/2016/508	17/11/2016 and 04/11/2016
**NK**	Kano State Hospitals Management Board	Kenneth Iregbu	8/10/1437AH	13/07/2016
**NN**	Health Research Ethics Committee (HREC), National Hospital, Abuja	Kenneth Iregbu	NHA/EC/017/2015	27/04/2015
**NW**	Health Research Ethics Committee (HREC), National Hospital, Abuja	Kenneth Iregbu	NHA/EC/017/2015	27/04/2015
**PC**	Shaheed Zulfiqar Ali Bhutto Medical University, Pakistan Institute of Medical Sciences (PIMS) Islamabad	Rabaab Zahra	NA, signed letter from Prof. Tabish Hazir	27/05/2015
**PP**	Shaheed Zulfiqar Ali Bhutto Medical University, Pakistan Institute of Medical Sciences (PIMS) Islamabad	Rabaab Zahra	NA, signed letter from Prof. Tabish Hazir	27/05/2015
**RK**	Republic of Rwanda, National Ethics Committee	Jean-Baptiste Mazarati	No342/RNEC/2015	10/11/2015
**RU**	Republic of Rwanda, National Ethics Committee	Jean-Baptiste Mazarati	No342/RNEC/2015	10/11/2015
**ZAT**	Stellenbosch University and Tygerberg Hospital, Research projects, Western Cape Government	Shaheen Mehtar	N15/07/063	04/12/2015 and 02/02/2016

Supplementary Table 4: **Questionnaire**

**Question**	**Responses**
Ward name or number	
Bed number or name	
Type of ward	Accident and emergency (A&E)
	Cardiology
	Critical care
	General surgery
	Gynaecology
	Maternity departments
	Neonatal unit
	Obstetrics and gynaecology units
	Sexual health (genitourinary medicine)
	Other
If other was selected, please specify	
Number of beds in this ward	
Number of bathrooms on this ward	
Where is the Mother situated on the ward?	First 1/3 (closest to door)
	Middle 1/3
	Last 1/3 (furthest from door)
	Side Room
	Other
If other was selected, please specify	
How many beds are in the side room?	
Age of the Mother in years (if not known please write "unknown" and an estimation)	
Date of birth of Mother if known	Date format: **DD/MM/YYYY**
Is this the Mothers first pregnancy?	Yes or No
Number of previous pregnancies	
Number, age and gender of living children	
Has the Mother had any multiple births?	No
	Twins (2)
	Triplets (3)
	Quadruplets (4)
	Quintuplets (5)
	Sextuplets (6)
	Septuplets (7)
	Octuplets (8)
	Nonuplets (9)
	Decuplets (10)
Please detail ages and genders:	
Number of miscarriages (option to bypass)	
Number of abortions (option to bypass)	
Number of stillbirths (option to bypass)	
Number of deceased children (option to bypass)	
Please provide details of deceased children, ages, gender, cause of death.	
In the past three months has the Mother suffered with any of these illnesses / conditions?	Diabetes
	Hypertension or cardiovascular disease
	Immune-compromised (Cancer, HIV, chronic liver diseases, use of steroids)
	TB

Question	Responses
	Malaria
	None
	Other
If other was selected, please specify	
Has the Mother received TB therapy?	Yes or No
In the three months prior to enrolment has the Mother attended a private healthcare clinic?	Yes or No
In the three months prior to enrolment has the Mother visited a traditional healer?	Yes or No
In the 12 months prior to enrolment has the mother travelled outside of the city, province, or country?	Yes or No
If yes please specify where?	
In the 12 months prior to enrolment has a household member travelled outside the city, province or country?	Yes or No
Please specify where	
In the 12 months prior to enrolment has the Mother been hospitalised?	Yes or No
Details on reason, duration, hospital	
In the three months prior to enrolment has the Mother used antibiotics (Oral or IV)?	Yes or No
Name of antibiotics used	Amikacin, Amoxicillin, Ampicillin, Aztreonam, Azithromycin, Carbenicillin, Cefaclor, Cefadroxil (cefadroxyl), Cefalexin (cephalexin), Cefaloridine (cephaloradine), Cefamandole, Cefazolin (cephazolin), Cefditoren, Cefepime, Cefixime, Cefotaxime, Cefotetan, Cefoperazone, Cefoxitin, Cefpodoxime, Cefradine (cephradine), Ceftaroline, Ceftazidime, Ceftibuten, Ceftiolene, Ceftizoxime, Ceftobiprole, Ceftriaxone, Cefuroxime, Chloramphenicol, Ciprofloxacin, Clarithromycin, Clindamycin,Cycloserine, Doripenem, Doxycycline, Ertapenem, Erythromycin, Flucloxacillin, Fosfomycin, Gentamicin, Imipenem, Kanamycin, Levofloxacin, Lincomycin, Linezolid, Meropenem, Metronidazole, Minocycline, Moxifloxacin, Nalidixic acid, Neomycin, Nitrofurantoin, Norfloxacin, Ofloxacin, Oxacillin, Oxytetracycline, Penicillin G, Piperacillin, Polymyxin B, Pristinamycin, Quinupristin/dalfopristin, Rifabutin, Rifampin, Streptomycin, Sulfamethoxazole, Telithromycin, Teicoplanin, Tetracycline, Ticarcillin, Tigecycline, Tobramycin, Trimethoprim-Sulfamethoxazole, Vancomycin, Unknown, Other
If other was selected, please specify:	
Overall household income per month (For modelling purposes, we converted this to multiples above and below the local area average household income per month)	20
	$20 - $30
	$30 - $40
	$40 - $50
	$50 - $100
	$100 - $250
	$250 - $500
	$500 - $1000
	$1,000 - $2000
What is the educational status of the Mother?	None
	Can read and write

Question	Responses
	Primary school
	Secondary school
	College or A-Levels
	Undergraduate
	Graduate
	Postgraduate
How would the Mother describe the residential area she lives in	Rural
	Urban
	Semi-rural
	Other
If other was selected, please specify	
What type of residence does the Mother live in?	Apartment
	Separate house
	Shack
	Homeless
	Other
If other was selected, please specify	
Number of bedrooms in home	
Number of people residing in the home	
What is the primary source of drinking water for the household?	Municipal network
	Water vendor (tanker)
	Private well
	Communal taps
	Other
If other was selected, please specify	
Is the drinking water boiled, filtered or untreated?	Boiled
	Filtered
	Neither
What is the primary water source used for?	Domestic water
	Drinking water
	Both
How many hours per day does the household have running water?	Scale: No supply to >12 hours
	Access via communal taps
	Other
If other was selected, please specify	
How many days per week does the household have running water?	Scale: No supply to continuous
Is there a solid waste pipe near the Mother's home (proximity of 100m)?	Yes or No
How frequently is solid waste collected?	Once a week or more
	Every 2 weeks
	Every 2 months
	We deal with it ourselves
	Other
If other was selected, please specify	
What sort of toilet does the Mother have within her home?	Sit down with flush
	Squat with flush
	Pit latrine
	Other
If other was selected, please specify	

Question	Responses
Is there stagnant or sewerage water near the Mother's house (proximity of 100m)?	Yes or No
Is the house served by a wastewater network?	Yes or No
Generally, does the Mother have access to soap?	Yes or No or Sometimes
How many times per day does the Mother generally wash her hands?	Scale: <1 to >5
How many days per week does the Mother generally take a shower or bath?	Scale: <1 to everyday
How many hours per day does the household have an electricity supply?	Scale: No supply to continuous
How many days per week does the household have an electricity supply?	Scale: No supply to continuous
Infant DOB	Date format: **DD/MM/YYYY**
Time of birth	HH:MM
Place of birth	Hospital
	Healthcare Centre or Clinic
	Home
	Other
Infant age group at admission	Less than 7 days old
	More than 7 days old
Gestational status	Pre-term
	Term
	Post-date
	Unknown
Gestational age (weeks)	
Premature rupture of membranes	Yes or No
Please provide details if possible	
How many hours after the waters broke until delivery	
Was the baby delivered by Caesarean Section	Yes or No
Type of C-section	Emergency
	Planned
	Unknown
Was the birth assisted in any other way	Yes or No
Please provide details e.g. forcep delivery, episiotomy	
Was this a breech delivery?	Yes or No
Perinatal asphyxia	Yes or No
Infant gender	Female or Male
Any other information in relation to the birth? (outcome or comments)	

Supplementary Table 5: Standard operating procedures

Overall link to standard operating procedures (SOPs) and methodology for BARNARDS	https://www.ineosoxford.ox.ac.uk/research/barnards
Appendix E	Ethics template
Appendix C	Consent form template
Appendix N	Neonatal sepsis clinical presentation worksheet
Appendix P	Phlebotomy checklist
Appendix M	Microbiology standard operating procedures at clinical sites
Appendix F	Follow up SOP

Supplementary Table 6: Suspected bacterial contaminants and frequency of their occurrence which led to removal from the outcome "laboratory-confirmed sepsis"

Excluded	Frequency
Arthrobacter oxydans	0
Bacillus megaterium	0
Bacillus pumilus	4
Bacillus sp.	0
Bacillus subtilis	1
Cellulosimicrobium cellulans	1
Corynebacterium amycolatum	0
Corynebacterium freneyi	0
Kocuria marina	1
Kocuria palustris	0
Lysinibacillus boronitolerans	0
Microbacterium arborescens	1
Microbacterium barkeri	0
Microbacterium paraoxydans	1
Microbacterium testaceum	0
Micrococcus luteus	4
Paenibacillus amylolyticus	0
Staphylococcus arlettae	1
Staphylococcus capitis	1
Staphylococcus caprae	1
Staphylococcus cohnii	1
Staphylococcus equorum	1
Staphylococcus gallinarum	2
Staphylococcus hominis	7
Staphylococcus saprophyticus	3
Staphylococcus succinus	0
Staphylococcus warneri	1
Staphylococcus xylosus	0
Streptomyces violaceoruber	0

To estimate the association between birthweight and laboratory-confirmed sepsis after adjusting for known and measured confounders, we fitted a multivariable Poisson regression with robust standard errors, with pre-term birth as the exposure, laboratory-confirmed sepsis as the outcome, and the following confounders (as suggested by the minimal sufficient adjustment set implied in our Directed Acyclic Graph **(**DAG) below): birth as part of a multiple, maternal hypertension, maternal age, maternal infection in the three months prior to enrolment, parity, premature rupture of membranes, and markers of socioeconomic status including type of residence, type of toilet in home, primary source of drinking water, overall household income per month, and electricity supply in home.

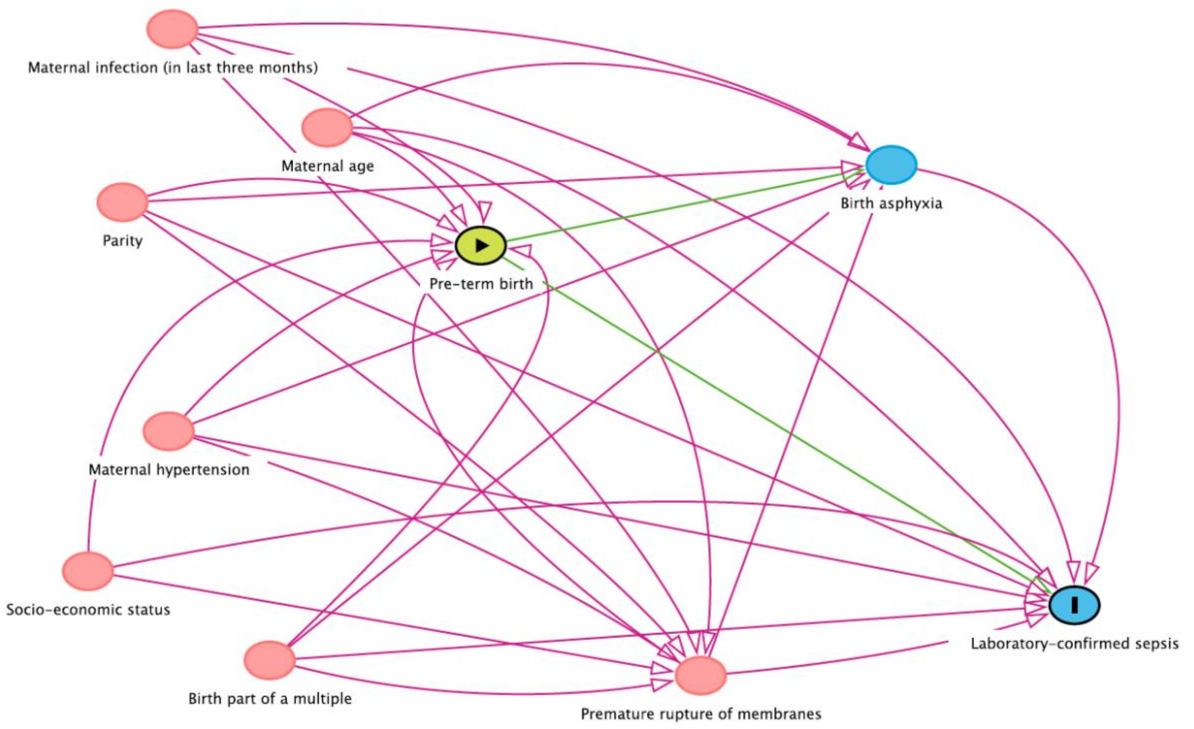

Supplementary Figure 2: Directed Acyclic Graph illustrating the pathways from pre-term birth (<37 weeks' gestation) (exposure) to laboratory-confirmed sepsis (outcome)

To estimate the association between premature rupture of membranes and laboratory-confirmed sepsis after adjusting for known and measured confounders, we fitted a multivariable Poisson regression with robust standard errors, with premature rupture of membranes as the exposure, laboratory-confirmed sepsis as the outcome, and the following confounders: birth as part of a multiple, maternal age, maternal hypertension, maternal infection in the three months prior to enrolment, parity, and markers of socioeconomic status (as suggested by the minimal sufficient adjustment set implied in our DAG below). We extended this model to include a pre-term birth x premature rupture of membranes interaction term to explore whether the association between premature rupture of membranes and laboratory-confirmed sepsis differed depending on whether the neonate was born at less than 37-weeks' gestation.

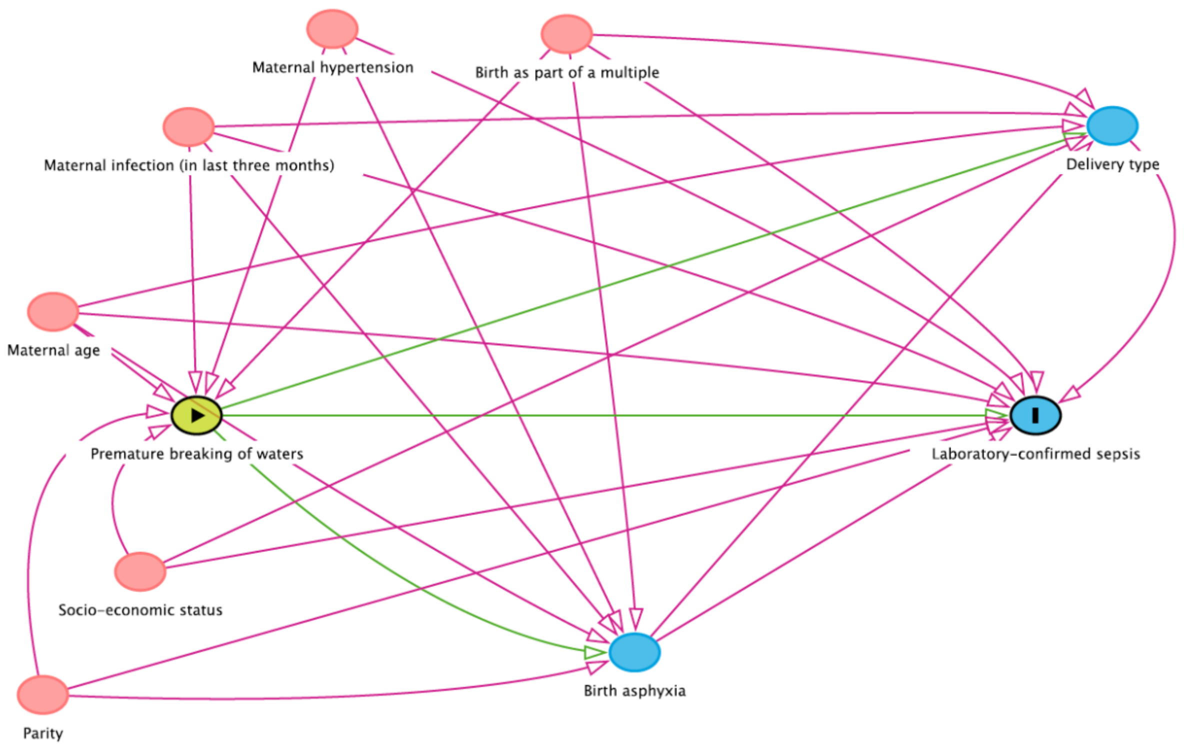

Supplementary Figure 3: Directed Acyclic Graph illustrating the pathways from premature rupture of membranes (exposure) to laboratory-confirmed sepsis (outcome)

To estimate the association between delivery type and laboratory-confirmed sepsis after adjusting for known and measured confounders, we fitted a multivariable Poisson regression with robust standard errors, with delivery type as the exposure, laboratory-confirmed sepsis as the outcome, and the following confounders: birth as part of a multiple, birth asphyxia, maternal age, maternal infection in the three months prior to enrolment, parity, premature rupture of membranes, and markers of socioeconomic status (as suggested by the minimal sufficient adjustment set implied in our DAG below).

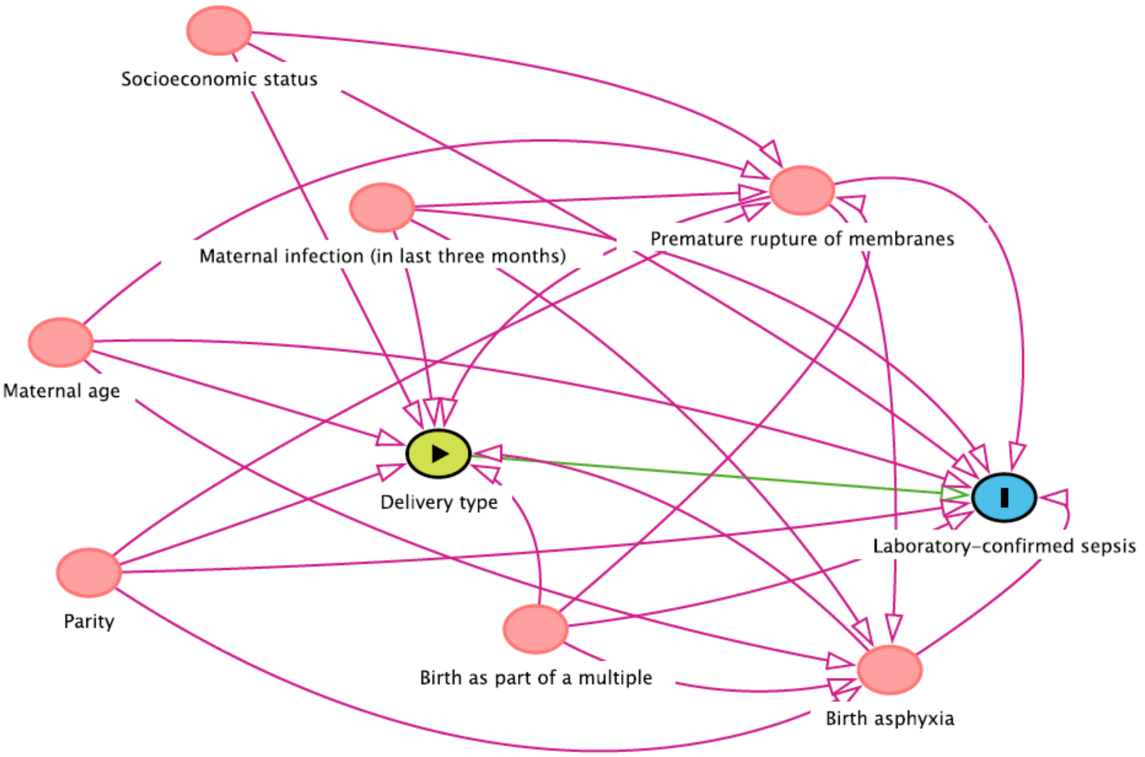

Supplementary Figure 4: Directed Acyclic Graph illustrating the pathways from delivery type (exposure) to laboratory-confirmed sepsis (outcome)

To estimate the association between delivery type and laboratory-confirmed sepsis after adjusting for known and measured confounders, we fitted a multivariable Poisson regression with robust standard errors, with delivery type as the exposure, laboratory-confirmed sepsis as the outcome, and the following confounders: birth as part of a multiple, birthweight, maternal age, maternal hypertension, maternal infection in the three months prior to enrolment, parity, pre-term delivery, and premature rupture of membranes (as suggested by the minimal sufficient adjustment set implied in our DAG, below).

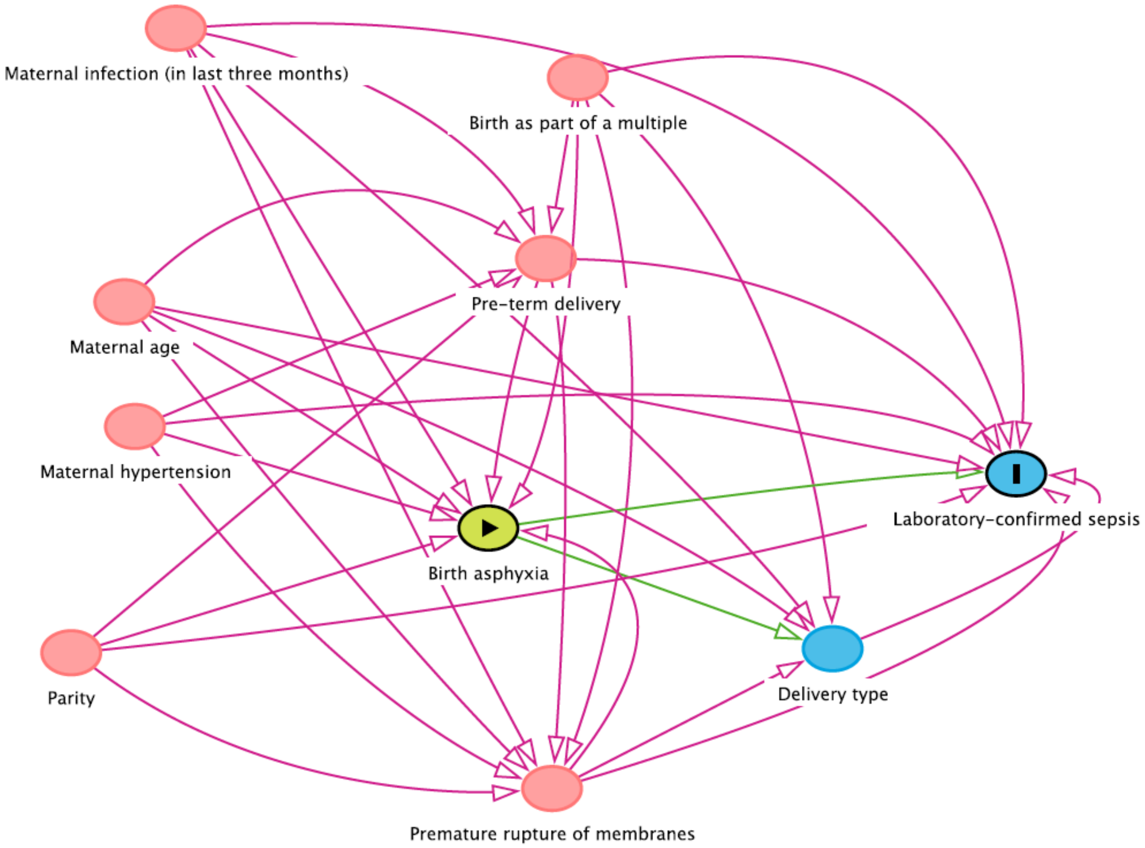

Supplementary Figure 5: Directed Acyclic Graph illustrating the pathways from birth asphyxia (exposure) to laboratory-confirmed sepsis (outcome)

Supplementary Table 7: Missing Data

Variable	Missing (n)	Observed (n)	Total neonates (n)	% Missing
Mother's age category	0	30557	30557	0
Diabetes	0	30557	30557	0
Hypertension	0	30557	30557	0
Immune-compromised	0	30557	30557	0
Malaria	0	30557	30557	0
TB	0	30557	30557	0
Infection	0	30557	30557	0
Other illness	0	30557	30557	0
Typhoid	0	30557	30557	0
Member of household travelled outside city, province or country in previous 12 months	0	30557	30557	0
Household income	0	30557	30557	0
Number of beds in residence	0	30557	30557	0
Number of people in residence	0	30557	30557	0
Current birth part of a multiple	0	30557	30557	0
Type of ward	3	30554	30557	0·01
Bathroom on the ward	3	30554	30557	0·01
Number of beds on the ward	7	30550	30557	0·02
Perinatal asphyxia	7	30550	30557	0·02
Mother visited a traditional healer in previous three months	8	30549	30557	0·03
Frequency of solid waste collection, if there is solid waste near	9	30548	30557	0·03
Pregnancy history	10	30547	30557	0·03
Type of toilet in household	10	30547	30557	0·03
Handwashing frequency	12	30545	30557	0·04
Presence of stagnant or sewage water near home	12	30545	30557	0·04

Variable	Missing (n)	Observed (n)	Total neonates (n)	% Missing
Is the water treated	13	30544	30557	0·04
Mother has access to soap	14	30543	30557	0·05
Whether house is served by wastewater network	15	30542	30557	0·05
Description of residential area	21	30536	30557	0·07
Type of residence	21	30536	30557	0·07
Received private healthcare in previous three months	30	30527	30557	0·10
Electricity supply in household	52	30505	30557	0·17
Breech presentation	57	30500	30557	0·19
Delivery type	59	30498	30557	0·19
Mother's highest level of education	112	30445	30557	0·37
Primary source of drinking water	144	30413	30557	0·47
Mother hospitalised in previous 12 months	503	30054	30557	1·65
Premature rupture of membranes	621	29936	30557	2·03
Mother used antibiotics in previous three months	689	29868	30557	2·25
Gestational age	1540	29017	30557	5·04
Mother's location on the ward	2445	28112	30557	8·00
Mother's shower or bath frequency	12382	18175	30557	40·52

Supplementary Tables 8a 8b: Descriptive Data*

SITE ID			BC	BK	ES	IN	NK	NN	NW	PC	PP	RU	RK	ZAT	Total
Cohort size	Mother	Count	550	1374	4005	1077	5339	1440	2156	402	7071	1141	1981	2947	29483
		%	1·87	4·66	13·58	3·65	18·11	4·88	7·31	1·36	23·98	3·87	6·72	10·00	100·00
	Baby	Count	563	1386	4187	1126	5584	1531	2224	415	7197	1173	2005	3166	30557
		%	1·84	4·54	13·70	3·68	18·27	5·01	7·28	1·36	23·55	3·84	6·56	10·36	100·00

Domain	Characteristic	Categories		BC	BK	ES	IN	NK	NN	NW	PC	PP	RU	RK	ZAT	Total
Healthcare	Type of ward (n=29483 mothers)	Maternity	Count	0	0	0	1076	3537	521	1114	7	3	1109	1765	11	9143
			%	0·00	0·00	0·00	99·91	66·25	36·18	51·67	1·74	0·04	97·20	89·09	0·37	31·01
		Neonatal	Count	145	12	36	0	41	158	13	80	1834	24	214	146	2703
			%	26·36	0·87	0·90	0·00	0·77	10·97	0·60	19·90	25·94	2·10	10·80	4·95	9·17
		Obstetrics & Gynaecology	Count	402	1358	1982	0	1758	755	1009	293	5227	1	0	2789	15574
			%	73·09	98·84	49·49	0·00	32·93	52·43	46·80	72·89	73·92	0·09	0·00	94·64	52·82
		Others	Count	3	3	1987	1	3	6	19	20	6	6	2	1	2057
			%	0·55	0·22	49·61	0·09	0·06	0·42	0·88	4·98	0·08	0·53	0·10	0·03	6·98
		Unknown	Count	0	1	0	0	0	0	1	2	1	1	0	0	6
			%	0·00	0·07	0·00	0·00	0·00	0·00	0·05	0·50	0·01	0·09	0·00	0·00	0·02
	Number of beds in the ward (n=29476 mothers)	1 to 3	Count	0	2	0	0	12	19	86	46	161	437	49	2	814
			%	0·00	0·15	0·00	0·00	0·22	1·32	3·99	11·59	2·28	38·30	2·47	0·07	2·76
		4 to 10	Count	2	0	1987	0	13	1072	1278	333	6713	687	1721	20	13826
			%	0·36	0·00	49·61	0·00	0·24	74·44	59·30	83·88	94·95	60·21	86·88	0 .68	46·91
		11 to 20	Count	74	5	1982	1054	436	307	791	15	195	1	211	52	5123
			%	13·45	0·36	49·49	97·86	8·17	21·32	36·71	3·78	2·76	0·09	10·65	1·76	17·38
		21+	Count	474	1367	36	23	4878	42	0	3	1	16	0	2873	9713
			%	86·18	99·49	0·90	2·14	91·37	2·92	0·00	0·76	0·01	1·40	0·00	97·49	32·95
	Any bathroom in the ward (n=29480 mothers)	0	Count	4	0	4005	0	6	3	1	6	177	420	630	19	5271
			%	0·73	0·00	100·00	0·00	0·11	0·21	0·05	1·49	2·50	36·81	31·80	0·64	17·88
		1 to 2	Count	140	1	0	3	3701	1181	2151	392	6873	492	8	2927	17869
			%	25·45	0·07	0·00	0·28	69·31	82·01	99·81	97·51	97·23	43·12	0·40	99·31	60·61
		3 to 4	Count	0	16	0	1070	1632	100	3	4	17	209	25	1	3077
			%	0·00	1·16	0·00	99·34	30·57	6·94	0·14	1·00	0·24	18·32	1·26	0·03	10·44
		5+	Count	406	1357	0	4	0	156	0	0	2	20	1318	0	3263
			%	73·81	98·76	0·00	0·37	0·00	10·83	0·00	0·00	0·03	1·75	66·53	0·00	11·07

Domain	Characteristic	Categories		BC	BK	ES	IN	NK	NN	NW	PC	PP	RU	RK	ZAT	Total
	Mother location on the ward (n=29483 mothers)	First 1/3 (closest to door)	Count	257	394	1072	279	2002	609	932	227	2993	609	106	29	9509
			%	46·73	28·68	26·77	25·91	37·50	42·29	43·23	56·47	42·33	53·37	5·35	0·98	32·25
		Middle 1/3	Count	249	725	1940	312	1803	418	583	99	2194	277	1294	24	9918
			%	45·27	52·77	48·44	28·97	33·77	29·03	27·04	24·63	31·03	24·28	65·31	0 .81	33·64
		Last 1/3 (furthest from door)	Count	44	245	950	315	1498	349	618	67	1751	248	12	27	6124
			%	8·00	17·83	23·72	29·25	28·06	24·24	28·66	16·67	24·76	21·74	0·61	0·92	20·77
		Side Room	Count	0	0	0	2	2	5	0	1	42	1	253	1029	1335
			%	0·00	0·00	0·00	0·19	0·04	0·35	0·00	0·25	0·59	0·09	12·77	34·92	4·53
		Outpatient	Count	0	0	0	0	11	0	0	0	0	0	0	0	11
			%	0·00	0·00	0·00	0·00	0·21	0·00	0·00	0·00	0·00	0·00	0·00	0·00	0·04
		Unknown	Count	0	10	43	169	23	59	23	8	91	6	316	1838	2586
			%	0·00	0·73	1·07	15·69	0·43	4·10	1·07	1·99	1·29	0·53	15·95	62·37	8·77
Maternal	Mother's estimated age (n=29483 mothers)	<=20	Count	131	488	466	171	1211	21	77	30	712	33	188	259	3787
			%	23·82	35·52	11·64	15·88	22·68	1·46	3·57	7·46	10·07	2·89	9·49	8·78	12·84
		21-35	Count	416	863	3296	856	3519	1148	1779	351	5905	915	1470	2160	22678
			%	75·64	62·81	82·30	79·48	65·91	79·72	82·51	87·31	83·51	80·19	74·20	73·29	76·92
		>35	Count	3	23	243	50	609	271	300	21	454	193	323	528	3018
			%	0·55	1·67	6·07	4·64	11·41	18·82	13·91	5·22	6·42	16·91	16·30	17·92	10·24
	Number of previous pregnancy (n=29470 mothers)	No previous pregnancy	Count	263	604	1748	534	1333	347	545	128	2523	452	773	629	9879
			%	47·82	43·96	43·78	49·58	24·97	24·10	25·28	31·84	35·69	39·61	39·02	21·34	33·52
		1	Count	155	435	1068	330	787	329	384	84	1454	220	505	671	6422
			%	28·18	31·66	26·75	30·64	14·74	22·85	17·81	20·90	20·57	19·28	25·49	22·77	21·79
		2	Count	93	215	630	156	615	272	470	74	1229	167	363	853	5137
			%	16·91	15·65	15·78	14·48	11·52	18·89	21·80	18·41	17·38	14·64	18·32	28·94	17·43
		3	Count	28	97	312	42	509	208	341	50	827	143	160	480	3197
			%	5·09	7·06	7·81	3·90	9·52	14·44	15·82	12·44	11·70	12·53	8·08	16·29	10·85
		4	Count	8	15	143	13	384	138	223	38	505	82	84	193	1826
			%	1·45	1·09	3·58	1·21	7·19	9·58	10·34	9·44	7·14	7·19	4·24	6·55	6·20
		5	Count	3	6	44	1	375	72	97	16	255	32	52	79	1032
			%	0·55	0·44	1·10	0·09	7·02	5·00	4·50	3·98	3·61	2·80	2·62	2·68	3·50
		6	Count	0	2	27	0	275	41	48	3	139	27	22	27	611
			%	0·00	0·15	0·68	0·00	5·15	2·85	2·23	0·75	1·97	2·37	1·11	0·92	2·07
		7	Count	0	0	11	1	284	14	20	5	71	6	14	11	437
			%	0·00	0·00	0·28	0·09	5·32	0·97	0 .93	1·24	1·00	0·53	0·71	0·37	1·48
		8	Count	0	0	5	0	224	10	13	2	37	4	4	2	301
			%	0·00	0·00	0·13	0·00	4·20	0·69	0·60	0·50	0·52	0·35	0·20	0·07	1·02
		9	Count	0	0	3	0	179	5	5	1	9	3	4	1	210

Domain	Characteristic	Categories		BC	BK	ES	IN	NK	NN	NW	PC	PP	RU	RK	ZAT	Total
			%	0·00	0·00	0·08	0·00	3·35	0·35	0·23	0·25	0·13	0·26	0·20	0·03	0·71
		10	Count	0	0	2	0	106	2	4	1	8	1	0	1	125
			%	0·00	0·00	0·05	0·00	1·99	0·14	0·19	0·25	0·11	0·09	0·00	0·03	0·42
		>10	Count	0	0	0	0	268	2	6	0	13	4	0	0	293
			%	0·00	0·00	0·00	0·00	5·02	0·14	0·28	0·00	0·18	0·35	0·00	0·00	0·99
	Any previous multiple birth (n=29440 mothers)	No previous pregnancy	Count	263	604	1748	534	1333	347	545	128	2523	452	773	629	9879
			%	47·82	43·96	43·96	49·58	24·97	24·10	25·28	32·65	35·70	39·61	39·02	21·34	33·56
		No	Count	287	765	2200	543	3867	1068	1548	261	4453	669	1191	2272	19124
			%	52·18	55·68	55·33	50·42	72·44	74·17	71·80	66·58	63·00	58·63	60·12	77·09	64·95
		Yes	Count	0	5	28	0	138	25	63	3	92	20	17	46	437
			%	0·00	0·36	0·70	0·00	2·59	1·74	2·92	0·77	1·30	1·75	0·86	1·56	1·48
	Number of previous miscarriages (n=27648 mothers)	No previous pregnancy	Count	263	604	1748	534	1333	347	545	128	2523	452	773	629	9879
			%	47·82	43·96	80·11	49·58	24·97	24·10	25·28	32·49	35·70	39·61	39·02	21·34	35·73
		0	Count	267	671	5	342	2486	710	1197	186	2996	681	1206	1735	12482
			%	48·55	48·84	0·23	31·75	46·56	49·31	55·52	47·21	42·39	59·68	60·88	58·87	45·15
		1	Count	17	93	364	165	950	258	294	58	1013	5	1	456	3674
			%	3·09	6·77	16·68	15·32	17·79	17·92	13·64	14·72	14·33	0·44	0·05	15·47	13·29
		2	Count	2	3	52	27	339	80	84	12	355	1	1	82	1038
			%	0·36	0·22	2·38	2·51	6·35	5·56	3·90	3·05	5·02	0·09	0·05	2·78	3·75
		3	Count	1	2	12	7	135	26	26	9	97	1	0	28	344
			%	0·18	0·15	0·55	0·65	2·53	1·81	1·21	2·28	1·37	0·09	0·00	0·95	1·24
		4	Count	0	1	0	2	54	12	7	0	43	1	0	13	133
			%	0·00	0·07	0·00	0·19	1·01	0·83	0·32	0·00	0·61	0·09	0·00	0·44	0·48
		5	Count	0	0	0	0	23	4	1	1	16	0	0	1	46
			%	0·00	0·00	0·00	0·00	0·43	0·28	0·05	0·25	0·23	0·00	0·00	0·03	0·17
		6	Count	0	0	0	0	6	1	0	0	3	0	0	1	11
			%	0·00	0·00	0·00	0·00	0·11	0·07	0·00	0·00	0·04	0·00	0·00	0·03	0·04
		7	Count	0	0	0	0	6	0	1	0	3	0	0	1	11
			%	0·00	0·00	0·00	0·00	0·11	0·00	0·05	0·00	0·04	0·00	0·00	0·03	0·04
		8	Count	0	0	0	0	5	1	0	0	1	0	0	0	7
			%	0·00	0·00	0·00	0·00	0·09	0·07	0·00	0·00	0·01	0·00	0·00	0·00	0·03
		9	Count	0	0	1	0	0	0	0	0	2	0	0	0	3
			%	0·00	0·00	0·05	0·00	0·00	0·00	0·00	0·00	0·03	0·00	0·00	0·00	0·01
		10	Count	0	0	0	0	2	1	1	0	1	0	0	1	6
			%	0·00	0·00	0·00	0·00	0·04	0·07	0·05	0·00	0·01	0·00	0·00	0·03	0·02
		>10	Count	0	0	0	0	0	0	0	0	14	0	0	0	14
			%	0·00	0·00	0·00	0·00	0·00	0·00	0·00	0·00	0·20	0·00	0·00	0·00	0·05

Domain	Characteristic	Categories		BC	BK	ES	IN	NK	NN	NW	PC	PP	RU	RK	ZAT	Total
	Number of previous abortions (n=27635 mothers)	No previous pregnancy	Count	263	604	1748	534	1333	347	545	128	2523	452	773	629	9879
			%	47·82	43·96	79·78	49·58	24·97	24·10	25·28	32·74	35·70	39·61	39·02	21·34	35·72
		0	Count	222	637	3	474	3972	857	1310	250	4432	521	1060	2155	15893
			%	40·36	46·36	0·14	44·01	74·40	59·51	60·76	63·94	62·71	45·66	53·51	73·13	57·47
		1	Count	53	107	368	58	27	113	178	7	69	108	118	149	1355
			%	9·64	7·79	16·80	5·39	0·51	7·85	8·26	1·79	0·98	9·47	5·96	5·06	4·90
		2	Count	11	19	58	8	3	70	73	5	21	35	23	11	337
			%	2·00	1·38	2·65	0·74	0·06	4·86	3·39	1·28	0·30	3·07	1·16	0·37	1·22
		3	Count	1	5	11	2	2	35	36	1	3	13	6	2	117
			%	0·18	0·36	0·50	0·19	0·04	2·43	1·67	0·26	0·04	1·14	0·30	0·07	0·42
		4	Count	0	2	2	0	1	12	8	0	1	7	0	0	33
			%	0·00	0·15	0·09	0·00	0·02	0·83	0·37	0·00	0·01	0·61	0·00	0·00	0·12
		5	Count	0	0	0	0	0	3	2	0	0	2	1	0	8
			%	0·00	0·00	0·00	0·00	0·00	0·21	0·09	0·00	0·00	0·18	0·05	0·00	0·03
		6	Count	0	0	0	1	0	2	1	0	0	0	0	0	4
			%	0·00	0·00	0·00	0·09	0·00	0·14	0·05	0·00	0·00	0·00	0·00	0·00	0·01
		7	Count	0	0	0	0	0	0	1	0	0	1	0	0	2
			%	0·00	0·00	0·00	0·00	0·00	0·00	0·05	0·00	0·00	0·09	0·00	0·00	0·01
		8	Count	0	0	0	0	0	1	0	0	0	1	0	0	2
			%	0·00	0·00	0·00	0·00	0·00	0·07	0·00	0·00	0·00	0·09	0·00	0·00	0·01
		9	Count	0	0	1	0	0	0	2	0	1	1	0	0	5
			%	0·00	0·00	0·05	0·00	0·00	0·00	0·09	0·00	0·01	0·09	0·00	0·00	0·02
		10	Count	0	0	0	0	0	0	0	0	0	0	0	0	0
			%	0·00	0·00	0·00	0·00	0·00	0·00	0·00	0·00	0·00	0·00	0·00	0·00	0·00
	Number of previous stillbirths (n=27365 mothers)	No previous pregnancy	Count	263	604	1748	534	1333	347	545	128	2523	452	773	629	9879
			%	47·82	43·96	91·18	49·58	24·97	24·10	25·28	32·74	35·70	39·61	39·02	21·34	36·08
		0	Count	277	727	0	536	3181	1044	1492	249	4265	648	1098	2094	15611
			%	50·36	52·91	0·00	49·77	59·58	72·50	69·20	63·68	60·35	56·79	55·43	71·06	57·02
		1	Count	9	41	151	7	620	44	106	11	222	35	97	206	1549
			%	1·64	2·98	7·88	0·65	11·61	3·06	4·92	2·81	3·14	3·07	4·90	6·99	5·66
		2	Count	1	2	14	0	142	5	10	3	32	3	11	14	237
			%	0·18	0·15	0·73	0·00	2·66	0·35	0·46	0·77	0·45	0·26	0 .56	0·48	0·87
		3	Count	0	0	3	0	35	0	2	0	4	2	1	4	51
			%	0·00	0·00	0·16	0·00	0·66	0·00	0·09	0·00	0·06	0·18	0·05	0·14	0·19
		4	Count	0	0	1	0	16	0	1	0	5	0	0	0	23
			%	0·00	0·00	0·05	0·00	0·30	0·00	0·05	0·00	0·07	0·00	0·00	0·00	0·08
		5	Count	0	0	0	0	7	0	0	0	1	1	0	0	9

Domain	Characteristic	Categories		BC	BK	ES	IN	NK	NN	NW	PC	PP	RU	RK	ZAT	Total
			%	0·00	0·00	0·00	0·00	0·13	0·00	0·00	0·00	0·01	0·09	0·00	0·00	0·03
		6	Count	0	0	0	0	3	0	0	0	0	0	1	0	4
			%	0·00	0·00	0·00	0·00	0·06	0·00	0·00	0·00	0·00	0·00	0·05	0·00	0·01
		7	Count	0	0	0	0	2	0	0	0	0	0	0	0	2
			%	0·00	0·00	0·00	0·00	0·04	0·00	0·00	0·00	0·00	0·00	0·00	0·00	0·01
		8	Count	0	0	0	0	0	0	0	0	0	0	0	0	0
			%	0·00	0·00	0·00	0·00	0·00	0·00	0·00	0·00	0·00	0·00	0·00	0·00	0·00
		9	Count	0	0	0	0	0	0	0	0	0	0	0	0	0
			%	0·00	0·00	0·00	0·00	0·00	0·00	0·00	0·00	0·00	0·00	0·00	0·00	0·00
	Number of previous deceased child (n=27352 mothers)	No previous pregnancy	Count	263	604	1748	534	1333	347	545	128	2523	452	773	629	9879
			%	47·82	43·96	93·03	49·58	24·97	24·10	25·28	31·92	35·70	39·61	39·02	21·34	36·12
		0	Count	273	722	2	516	2944	1007	1471	243	3913	644	1107	2163	15005
			%	49·64	52·55	0·11	47·91	55·14	69·93	68·23	60·60	55·37	56·44	55·88	73·40	54·86
		1	Count	13	43	119	24	674	76	122	22	468	39	88	145	1833
			%	2·36	3·13	6·33	2·23	12·62	5·28	5·66	5·49	6·62	3·42	4·44	4·92	6·70
		2	Count	1	4	10	2	230	10	14	6	111	3	10	9	410
			%	0·18	0·29	0·53	0·19	4·31	0·69	0·65	1·50	1·57	0·26	0·50	0·31	1·50
		3	Count	0	1	0	1	92	0	1	2	24	2	2	0	125
			%	0·00	0·07	0·00	0·09	1·72	0·00	0·05	0·50	0·34	0·18	0·10	0·00	0·46
		4	Count	0	0	0	0	40	0	2	0	10	0	1	1	54
			%	0·00	0·00	0·00	0·00	0·75	0·00	0·09	0·00	0·14	0·00	0·05	0·03	0·20
		5	Count	0	0	0	0	8	0	0	0	2	0	0	0	10
			%	0·00	0·00	0·00	0·00	0·15	0·00	0·00	0·00	0·03	0·00	0·00	0·00	0·04
		6	Count	0	0	0	0	9	0	1	0	1	1	0	0	12
			%	0·00	0·00	0·00	0·00	0·17	0·00	0·05	0·00	0·01	0·09	0·00	0·00	0·04
		7	Count	0	0	0	0	6	0	0	0	0	0	0	0	6
			%	0·00	0·00	0·00	0·00	0·11	0·00	0·00	0·00	0·00	0·00	0·00	0·00	0·02
		8	Count	0	0	0	0	0	0	0	0	1	0	0	0	1
			%	0·00	0·00	0·00	0·00	0·00	0·00	0·00	0·00	0·01	0·00	0·00	0·00	0·00
		9	Count	0	0	0	0	0	0	0	0	1	0	0	0	1
			%	0·00	0·00	0·00	0·00	0·00	0·00	0·00	0·00	0·01	0·00	0·00	0·00	0·00
		10	Count	0	0	0	0	1	0	0	0	0	0	0	0	1
			%	0·00	0·00	0·00	0·00	0·02	0·00	0·00	0·00	0·00	0·00	0·00	0·00	0·00
		>10	Count	0	0	0	0	2	0	0	0	13	0	0	0	15
			%	0·00	0·00	0·00	0·00	0·04	0·00	0·00	0·00	0·18	0·00	0·00	0·00	0·05
	Diabetes (prior three months) (n=29483 mothers)	No	Count	527	1345	3984	1021	5316	1416	2150	394	6906	1132	1979	2673	28843
			%	95·81	97·89	99·48	94·80	99·56	98·33	99·72	98·01	97·67	99·20	99·90	90·70	97·83

Domain	Characteristic	Categories		BC	BK	ES	IN	NK	NN	NW	PC	PP	RU	RK	ZAT	Total
		Yes	Count	23	29	21	56	23	24	6	8	165	9	2	274	640
			%	4·18	2·11	0·52	5·20	0·43	1·67	0·28	1·99	2·33	0·79	0·10	9·30	2·17
	Hypertension or Cardiovascular Disease (prior three months) (n=29483 mothers)	No	Count	517	1329	3913	1034	4985	1358	2111	382	6459	1055	1967	1914	27024
			%	94·00	96·72	97·70	96·01	93·37	94·31	97·91	95·02	91·34	92·45	99·29	64·95	91·66
		Yes	Count	33	45	92	43	354	82	45	20	612	86	14	1033	2459
			%	6·00	3·28	2·30	3·99	6·63	5·69	2·09	4·98	8·66	7·54	0·71	35·05	8·34
	Immune compromised (prior three months) (n=29483 mothers)	No	Count	545	1361	3938	1072	5300	1333	2045	398	6971	1131	1931	1940	27965
			%	99·09	99·05	98·33	99·54	99·27	92·56	94·84	99·00	98·59	99·12	97·48	65·83	94·84
		Yes	Count	5	13	67	5	39	107	111	4	100	10	50	1007	1518
			%	0·91	0·95	1·67	0·46	0·73	7·43	5·15	1·00	1·41	0·88	2·52	34·17	5·15
	Malaria (prior three months) (n=29483 mothers)	No	Count	550	1374	4005	1077	3849	895	1472	402	7059	1072	1819	2942	26516
			%	100·00	100·00	100·00	100·00	72·09	62·15	68·27	100·00	99·83	93·95	91·81	99·83	89·94
		Yes	Count	0	0	0	0	1490	545	684	0	12	69	162	5	2967
			%	0·00	0·00	0·00	0·00	27·91	37·85	31·73	0·00	0·17	6·05	8·18	0·17	10·06
	TB (prior three months) (n=29483 mothers)	No	Count	550	1374	4005	1077	5336	1440	2156	401	7063	1141	1979	2924	29446
			%	100·00	100·00	100·00	100·00	99·94	100·00	100·00	99·75	99·89	100·00	99·90	99·22	99·87
		Yes	Count	0	0	0	0	3	0	0	1	8	0	2	23	37
			%	0·00	0·00	0·00	0·00	0·06	0·00	0·00	0·25	0·11	0·00	0·10	0·78	0·13
	Infection (prior three months) (n=29483 mothers)	No	Count	526	1332	4002	1076	5233	1249	2139	396	7008	1085	1967	2941	28954
			%	95·64	96·94	99·93	99·91	98·01	86·74	99·20	98·51	99·11	95·09	99·29	99·80	98·20
		Yes	Count	24	42	3	1	106	191	17	6	63	56	14	6	529
			%	4·36	3·06	0·07	0·09	1·99	13·26	0·79	1·49	0·89	4·91	0·71	0·20	1·79
	Other illness (prior three months) (n=29483 mothers)	No	Count	75	106	232	95	1895	775	817	36	916	210	239	1854	7250
			%	13·64	7·71	5·79	8·82	35·49	53·82	37·89	8·96	12·95	18·40	12·06	62·91	24·59
		Yes	Count	475	1268	3773	982	3444	665	1339	366	6155	931	1742	1093	22233
			%	86·36	92·29	94·20	91·18	64·51	46·18	62·11	91·04	87·05	81·59	87·94	37·09	75·41
	Typhoid (prior three months) (n=29483 mothers)	No	Count	550	1374	4005	1077	5310	1399	2119	401	7059	1141	1981	2947	29363
			%	100·00	100·00	100·00	100·00	99·45	97·15	98·28	99·75	99·83	100·00	100·00	100·00	99·59
		Yes	Count	0	0	0	0	29	41	37	1	12	0	0	0	120
			%	0·00	0·00	0·00	0·00	0·54	2·85	1·72	0·25	0·17	0·00	0·00	0·00	0·41
	Received TB therapy (prior three months) (n=539 mothers)	No	Count	0	0	1	0	1	0	0	163	330	0	1	2	498
			%	0·00	0·00	25·00	0·00	33·33	0·00	0·00	99·39	96·20	.	50·00	8·69	92·39
		Yes	Count	0	0	3	0	1	0	0	1	2	0	1	21	29
			%	0·00	0·00	75·00	0·00	33·33	0·00	0·00	0·61	0·58	.	50·00	91·30	5·38
		Unknown	Count	0	0	0	0	1	0	0	0	11	0	0	0	12
			%	0·00	0·00	0·00	0·00	33·33	0·00	0·00	0·00	3·21	.	0·00	0·00	2·23
		No	Count	188	632	3904	983	4377	1077	1930	183	2050	742	1942	2833	20841

Domain	Characteristic	Categories		BC	BK	ES	IN	NK	NN	NW	PC	PP	RU	RK	ZAT	Total
	Received private healthcare in the prior 3 months (n=29454 mothers)		%	34·18	46·00	98·12	91·27	81·98	74·79	89·52	45·52	29·00	65·03	98·03	96·13	70·76
		Yes	Count	362	742	75	94	962	363	226	219	5018	399	39	114	8613
			%	65·81	54·00	1·88	8·73	18·02	25·21	10·48	54·48	71·00	34·97	1·97	3·87	29·24
	Visited traditional healer in the prior 3 months (n=29475 mothers)	No	Count	402	1259	3995	1067	2945	1395	2065	399	6956	1042	1832	2930	26287
			%	73·09	91·63	99·90	99·06	55·16	96·88	95·78	99·25	98·40	91·31	92·48	99·42	89·18
		Yes	Count	148	115	4	10	2394	45	91	3	113	99	149	17	3188
			%	26·91	8·36	0·10	0·93	44·84	3·13	4·22	0·75	1·60	8·68	7·52	0·58	10·82
	Hospitalised in the last 12 months (n=29001 mothers)	No	Count	542	1262	3420	1064	5104	1197	2061	397	6720	1020	1942	2642	27371
			%	98·55	91·84	97·02	98·79	95·59	83·13	95·59	98·76	95·06	89·40	98·03	89·65	94·38
		Yes	Count	3	112	19	13	200	240	93	5	334	115	38	303	1475
			%	0·55	8·15	0·54	1·21	3·75	16·67	4·31	1·24	4·72	10·08	1·92	10·28	5·09
		Unknown	Count	5	0	86	0	35	3	2	0	15	6	1	2	155
			%	0·91	0·00	2·44	0·00	0·66	0·21	0·09	0·00	0·21	0·53	0·05	0·07	0·53
	Used antibiotics in the prior 3 months (n=28836 mothers)	No	Count	528	1285	3914	1047	3675	1003	1753	399	6885	1009	1945	2392	25835
			%	98·69	93·59	98·64	97·67	73·65	72·42	83·95	99·75	98·47	90·33	98·23	81·47	89·59
		Yes	Count	7	88	54	25	1315	382	335	1	107	108	35	544	3001
			%	1·31	6·41	1·36	2·33	26·35	27·58	16·04	0·25	1·53	9·67	1·77	18·53	10·41
	Mother's educational status (n=29373 mothers)	None	Count	0	60	462	25	925	3	20	58	1341	9	97	18	3018
			%	0·00	4·37	11·85	2·32	17·33	0·21	0·93	14·43	18·97	0·79	4·90	0·61	10·27
		Limited	Count	86	348	1909	181	1160	31	108	72	1003	310	1498	144	6850
			%	15·64	25·33	48·97	16·81	21·73	2·15	5·01	17·91	14·19	27·17	75·62	4·89	23·32
		Secondary schooling	Count	444	864	1267	650	3130	436	1260	197	3319	555	340	2673	15135
			%	80·73	62·88	32·50	60·35	58·63	30·28	58·44	49·00	46·96	48·64	17·16	90·70	51·53
		University	Count	20	102	260	221	124	970	768	75	1405	267	46	112	4370
			%	3·64	7·42	6·67	20·52	2·32	67·36	35·62	18·66	19·88	23·40	2·32	3·80	14·88
	Is current birth a multiple? (n=29483 mothers)	No	Count	537	1362	3826	1028	5102	1366	2085	389	6959	1117	1957	2737	28465
			%	97·64	99·13	95·53	95·45	95·56	94·86	96·70	96·77	98·42	97·90	98·79	92·87	96·55
		Yes	Count	13	12	179	49	237	74	71	13	112	24	24	210	1018
			%	2·36	0·87	4·47	4·55	4·44	5·14	3·29	3·23	1·58	2·10	1·21	7·13	3·45
Living environment	Mother travelled outside (n=28919 mothers)	No	Count	545	1258	3040	1058	4525	907	1528	358	5375	1013	1943	1461	23011
			%	99·09	91·56	88·30	98·24	84·75	62·99	70·87	89·05	76·04	88·78	98·08	49·58	79·56
		Yes	Count	5	116	403	19	814	533	628	44	1694	128	38	1486	5908
			%	0·91	8·44	11·70	1·76	15·25	37·01	29·13	10·95	23·96	11·22	1·92	50·42	20·43
	Member of household travelled outside? (n=27863 mothers)	No	Count	484	977	2245	1059	3748	663	1244	344	5462	998	1920	2432	21576
			%	88·00	71·11	94·01	98·33	70·20	46·04	57·70	85·79	77·27	87·47	96·92	82·52	77·44
		Yes	Count	28	396	143	16	1531	771	902	56	1574	137	19	511	6084
			%	5·09	28·82	5·99	1·49	28·68	53·54	41·84	13·97	22·27	12·01	0·96	17·34	21·84

Domain	Characteristic	Categories		BC	BK	ES	IN	NK	NN	NW	PC	PP	RU	RK	ZAT	Total
		Don't know	Count	38	1	0	2	60	6	10	1	33	6	42	4	203
			%	6·91	0·07	0·00	0·19	1·12	0·42	0·46	0·25	0·47	0·53	2·12	0·14	0·73
	Overall household income per month (n=29483 mothers)	More than 20 times below average	Count	0	0	0	5	1818	81	1012	0	186	0	0	816	3918
			%	0·00	0·00	0·00	0·46	34·05	5·63	46·94	0·00	2·63	0·00	0·00	27·69	13·29
		Between 10 and 20 times below average	Count	0	0	42	163	780	287	372	0	393	357	0	166	2560
			%	0·00	0·00	1·05	15·13	14·61	19·93	17·25	0·00	5·56	31·29	0·00	5·63	8·68
		Up to 10 times below average	Count	12	284	2125	905	2738	1019	741	57	5705	698	1981	1878	18143
			%	2·18	20·67	53·06	84·03	51·28	70·76	34·37	14·18	80·68	61·17	100·00	63·73	61·54
		Average	Count	94	713	1160	4	2	53	31	137	391	83	0	87	2755
			%	17·09	51·89	28·96	0·37	0·04	3·68	1·44	34·08	5·53	7·27	0·00	2·95	9·34
		Up to 2 times above average	Count	57	314	425	0	1	0	0	39	58	3	0	0	897
			%	10·36	22·85	10·61	0·00	0·02	0·00	0·00	9·69	0·82	0·26	0·00	0·00	3·04
		More than 2 times above average	Count	387	63	253	0	0	0	0	169	338	0	0	0	1210
			%	70·36	4·59	6·32	0·00	0·00	0·00	0·00	42·04	4·78	0·00	0·00	0·00	4·10
	Mother's description from the residential area she lives in (n=29467 mothers)	Rural	Count	133	1207	482	438	296	22	383	187	1947	310	1729	1697	8831
			%	24·18	87·84	12·08	40·67	5·54	1·53	17·76	46·52	27·54	27·17	87·28	57·58	29·97
		Urban	Count	391	106	3019	591	3156	1319	1651	195	4939	763	82	1234	17446
			%	71·09	7·71	75·65	54·87	59·11	91·59	76·58	48·51	69·87	66·87	4·14	41·87	59·21
		Semi-rural	Count	26	61	490	48	1883	99	122	20	183	68	169	16	3185
			%	4·73	4·44	12·28	4·46	35·27	6·88	5·66	4·98	2·59	5·96	8·52	0·54	10·81
		Other	Count	0	0	0	0	4	0	0	0	0	0	1	0	5
			%	0·00	0·00	0·00	0·00	0·07	0·00	0·00	0·00	0·00	0·00	0·05	0·00	0·02
	Type of residence mother live in (n=29462 mothers)	Apartment	Count	306	35	5	41	2016	1275	2091	120	1778	12	5	205	7889
			%	55·64	2·55	0·13	3·81	37·76	88·54	96·99	29·85	25·15	1·05	0·25	6·96	26·78
		Separate house	Count	178	1337	3247	1012	2265	156	58	280	5249	1117	1973	1645	18517
			%	32·36	97·31	81·45	93·95	42·42	10·83	2·69	69·65	74·25	97·90	99·59	55·82	62·85
		Shack	Count	29	1	503	18	1050	9	4	1	22	11	2	1089	2739
			%	5·27	0·07	12·62	1·67	19·67	0·63	0·19	0·25	0·31	0·96	0·10	36·95	9·30
		Room	Count	0	0	0	1	0	0	2	0	3	0	0	1	7
			%	0·00	0·00	0·00	0·09	0·00	0·00	0·09	0·00	0·04	0·00	0·00	0·03	0·02
		Homeless	Count	0	1	9	1	6	0	0	1	4	1	1	0	24
			%	0·00	0·07	0·23	0·09	0·11	0·00	0·00	0·25	0·06	0·09	0·05	0·00	0·08
		Other	Count	37	0	222	4	2	0	1	0	13	0	0	7	286
			%	6·73	0·00	5·57	0·37	0·04	0·00	0·05	0·00	0·18	0·00	0·00	0·24	0·97
	Bedrooms in residence (n=29483 mothers)	0	Count	0	0	1206	1	1	2	2	1	12	22	0	3	1250
			%	0·00	0·00	30·11	0·09	0·02	0·14	0·09	0·25	0·17	1·93	0·00	0·10	4·24
		1	Count	75	204	2079	354	624	536	1000	59	811	67	413	973	7195

Domain	Characteristic	Categories		BC	BK	ES	IN	NK	NN	NW	PC	PP	RU	RK	ZAT	Total
			%	13·64	14·85	51·91	32·87	11·69	37·22	46·38	14·68	11·47	5·87	20·85	33·02	24·40
		2	Count	330	542	549	443	2004	593	816	114	2224	227	895	1339	10076
			%	60·00	39·45	13·71	41·13	37·54	41·18	37·85	28·36	31·45	19·89	45·18	45·44	34·18
		3	Count	97	319	103	145	1311	234	228	130	2171	469	478	483	6168
			%	17·64	23·22	2·57	13·46	24·56	16·25	10·58	32·34	30·70	41·10	24·13	16·39	20·92
		4+	Count	48	309	68	134	1399	75	110	98	1853	356	195	149	4794
			%	8·73	22·49	1·70	12·44	26·20	5·21	5·10	24·38	26·21	31·20	9·84	5·06	16·26
	Primary source of drinking water (n=29341 mothers)	Municipal network	Count	309	1	3372	504	971	236	317	126	1715	9	147	996	8703
			%	56·18	0·07	84·74	46·80	18·19	16·39	14·70	31·98	24·62	0·79	7·42	33·82	29·66
		Water vendor (tanker)	Count	0	14	13	19	1430	16	25	4	119	313	7	3	1963
			%	0·00	1·02	0·33	1·76	26·78	1·11	1·16	1·02	1·71	27·43	0·35	0·10	6·69
		Private well	Count	10	1343	147	72	1243	7	7	220	4249	571	5	9	7883
			%	1·82	97·74	3·69	6·69	23·28	0·49	0·32	55·84	61·01	50·04	0·25	0·31	26·87
		Communal taps	Count	58	16	401	87	727	164	236	23	567	247	1822	1929	6277
			%	10·55	1·16	10·08	8·08	13·62	11·39	10·95	5·84	8·14	21·65	91·97	65·50	21·39
		Sachet or Bottled Water	Count	0	0	1	18	795	977	1229	1	24	0	0	0	3045
			%	0·00	0·00	0·03	1·67	14·89	67·84	57·00	0·25	0·34	0·00	0·00	0·00	10·38
		Ground water	Count	173	0	45	377	173	40	342	20	291	1	0	8	1470
			%	31·45	0·00	1·13	35·00	3·24	2·78	15·86	5·08	4·18	0·09	0·00	0·27	5·01
	Water availability (hours per day) (n=29465 mothers)	No household supply	Count	183	1350	209	480	3247	251	465	249	4699	841	5	19	11998
			%	33·27	98·25	5·24	44·57	60·83	17·43	21·58	61·94	66·47	73·70	0·25	0·64	40·72
		Access to communal water supply	Count	58	16	401	88	959	233	988	23	578	250	1823	1931	7348
			%	10·55	1·16	10·05	8·17	17·97	16·18	45·85	5·72	8·18	21·91	92·02	65·52	24·94
		Intermittent	Count	0	0	1778	0	0	0	0	1	0	0	1	7	1787
			%	0·00	0·00	44·55	0·00	0·00	0·00	0·00	0·25	0·00	0·00	0·05	0·24	6·06
		<12 hrs	Count	61	1	405	507	799	152	131	51	807	7	152	23	3096
			%	11·09	0·07	10·15	47·08	14·97	10·56	6·08	12·69	11·42	0·61	7·67	0·78	10·51
		>12 hrs	Count	248	7	1198	2	333	804	571	78	985	43	0	967	5236
			%	45·09	0·51	30·02	0·19	6·24	55·83	26·50	19·40	13·93	3·77	0·00	32·81	17·77
	Water availability (days per week) (n=29464 mothers)	No household supply	Count	241	1366	610	568	4206	484	1453	272	5277	1091	1828	1950	19346
			%	43·82	99·42	15·29	52·74	78·79	33·61	67·42	67·66	74·65	95·62	92·28	66·17	65·66
		<=1 days	Count	3	0	472	1	285	2	1	11	20	2	4	6	807
			%	0·55	0·00	11·83	0·09	5·34	0·14	0·05	2·74	0·28	0·18	0·20	0·20	2·74
		2-3 days	Count	63	0	367	0	205	32	11	28	132	3	0	4	845
			%	11·45	0·00	9·19	0·00	3·84	2·22	0·51	6·97	1·87	0·26	0·00	0·14	2·87
		4-6 days	Count	189	4	2349	64	464	153	136	8	409	34	4	11	3825
			%	34·36	0·29	58·87	5·94	8·69	10·63	6·31	1·99	5·79	2·98	0·20	0·37	12·98

Domain	Characteristic	Categories		BC	BK	ES	IN	NK	NN	NW	PC	PP	RU	RK	ZAT	Total
		Continuous supply	Count	54	4	192	444	178	769	554	83	1231	11	145	976	4641
			%	9·82	0·29	4·81	41·23	3·33	53·40	25·71	20·65	17·41	0·96	7·32	33·12	15·75
	Is the drinking water treated (n=29470 mothers)	Boiled	Count	161	9	547	6	28	85	168	21	371	1083	1075	73	3627
			%	29·27	0·66	13·70	0·56	0·52	5·90	7·79	5·22	5·25	94·92	54·27	2·48	12·31
		Filtered	Count	178	15	0	186	517	45	65	64	1091	35	8	12	2216
			%	32·36	1·09	0·00	17·27	9·68	3·13	3·01	15·92	15·43	3·07	0·40	0·41	7·52
		Neither	Count	211	1350	3447	885	4794	1310	1923	317	5607	23	898	2862	23627
			%	38·36	98·25	86·30	82·17	89·79	90·97	89·19	78·86	79·31	2·02	45·33	97·12	80·17
	Primary source of water (n=29468 mothers)	Domestic	Count	59	1	99	24	887	505	294	17	372	945	1839	5	5047
			%	10·73	0·07	2·48	2·23	16·61	35·07	13·64	4·25	5·26	82·81	92·83	0·17	17·13
		Drinking	Count	33	6	3346	62	422	522	961	44	228	184	4	1	5813
			%	6·00	0·44	83·78	5·76	7·90	36·25	44·57	11·00	3·23	16·13	0·20	0·03	19·73
		Both	Count	458	1367	549	991	4030	413	901	339	6469	12	138	2941	18608
			%	83·27	99·49	13·75	92·01	75·48	28·68	41·79	84·75	91·51	1·05	6·97	99·8	63·15
	Frequency of solid waste collection if there is a pipe nearby (n=29474 mothers)	No solid waste pipe nearby	Count	396	103	2226	639	3980	855	1734	311	6059	900	1979	1300	20482
			%	72·00	7·50	55·69	59·33	74·55	59·38	80·43	77·36	85·70	78·88	99·90	44·11	69·49
		We deal with it ourselves	Count	46	1226	517	21	1151	86	201	22	419	2	1	46	3738
			%	8·35	89·23	12·93	1·95	21·56	5·97	9·32	5·47	5·93	0·18	0·05	1·56	12·68
		Once a week or more	Count	99	22	1032	367	20	379	178	50	389	67	1	1572	4176
			%	18·00	1·60	25·82	34·08	0·37	26·32	8·26	12·44	5·50	5·87	0·05	53·34	14·17
		Less than once a week	Count	8	23	222	17	159	120	42	5	63	172	0	8	839
			%	1·45	1·67	5·55	1·58	2·98	8·33	1·95	1·24	0·89	15·07	0·00	0·27	2·85
		Not disposed	Count	0	0	0	32	15	0	1	8	123	0	0	1	180
			%	0·00	0·00	0·00	2·97	0·28	0·00	0·05	1·99	1·74	0·00	0·00	0·03	0·61
		Other	Count	1	0	0	1	14	0	0	6	17	0	0	20	59
			%	0·18	0·00	0·00	0·09	0·26	0·00	0·00	1·49	0·24	0·00	0·00	0·68	0·20
	Toilet in home (n=29473 mothers)	No toilet	Count	0	0	932	0	1	3	9	0	9	0	0	88	1042
			%	0·00	0·00	23·32	0·00	0·02	0·21	0·42	0·00	0·13	0·00	0·00	2·99	3·54
		Sit down with flush	Count	227	0	252	245	347	1394	1830	64	659	347	27	2739	8131
			%	41·27	0·00	6·30	22·75	6·50	96·81	84·88	15·96	9·32	30·41	1·36	92·94	27·59
		Squat with flush	Count	61	5	219	831	2948	34	298	328	6300	67	6	14	11111
			%	11·09	0·36	5·48	77·16	55·22	2·36	13·82	81·80	89·11	5·87	0·30	0·48	37·70
		Pit latrine	Count	262	1369	2594	0	2003	9	19	1	25	727	1948	103	9060
			%	47·64	99·64	64·90	0·00	37·52	0·63	0·88	0·25	0·35	63·72	98·33	3·50	30·74
		Both sit down and squat	Count	0	0	0	0	40	0	0	6	61	0	0	0	107
			%	0·00	0·00	0·00	0·00	0·75	0·00	0·00	1·50	0·86	0·00	0·00	0·00	0·36
		Other	Count	0	0	0	1	0	0	0	2	16	0	0	3	22

Domain	Characteristic	Categories		BC	BK	ES	IN	NK	NN	NW	PC	PP	RU	RK	ZAT	Total
			%	0·00	0·00	0·00	0·09	0·00	0·00	0·00	0·50	0·23	0·00	0·00	0·10	0·07
	Presence of stagnant or sewage water near home (n=29471 mothers)	No	Count	471	1167	3891	1037	4035	1294	2021	289	6437	1006	1971	2648	26267
			%	85·64	84·93	97·42	96·29	75·58	89·86	93·74	71·89	91·05	88·17	99·50	89·84	89·13
		Yes	Count	79	207	103	40	1304	146	135	113	633	135	10	299	3204
			%	14·36	15·07	2·58	3·71	24·42	10·14	6·26	28·11	8·94	11·83	0·50	10·15	10·87
	Whether house is served by wastewater network (n=29468 mothers)	No	Count	230	1278	3543	43	1298	199	375	34	3503	1020	1974	432	13929
			%	41·82	93·01	88·75	3·99	24·31	13·82	17·39	8·48	49·55	89·40	99·65	14·66	47·27
		Yes	Count	320	96	449	1034	4041	1241	1781	367	3567	121	7	2515	15539
			%	58·18	6·99	11·25	96·01	75·69	86·18	82·61	91·52	50·45	10·60	0·35	85·34	52·73
	Electricity supply in household (hrs per day) (n=29440 mothers)	No supply	Count	6	86	191	0	415	7	12	0	20	195	1490	46	2468
			%	1·09	6·26	4·79	0·00	7·78	0·49	0·56	0·00	0·28	17·11	75·75	1·56	8·38
		<4 hrs	Count	10	0	43	1	720	224	62	6	86	23	5	2	1182
			%	1·82	0·00	1·08	0·09	13·49	15·57	2·88	1·49	1·22	2·02	0·25	0·07	4·01
		5-12 hrs	Count	79	17	1982	2	2868	706	1076	2	927	63	1	7	7730
			%	14·36	1·24	49·66	0·19	53·75	49·06	49·95	0·50	13·12	5·53	0·05	0·24	26·26
		>12 hrs	Count	455	1270	1775	1073	1333	502	1004	394	6032	859	471	2892	18060
			%	82·73	92·50	44·48	99·72	24·98	34·89	46·61	98·01	85·38	75·34	23·95	98·13	61·35
	Electricity supply in household (days per week) (n=29434 mothers)	No supply	Count	6	86	191	0	415	7	12	0	20	195	1490	46	2468
			%	1·09	6·26	4·79	0·00	7·78	0·49	0·56	0·00	0·28	17·11	75·75	1·56	8·38
		Less than half the week	Count	89	4	150	0	1797	436	165	0	161	29	4	9	2844
			%	16·18	0·29	3·76	0·00	33·68	30·30	7·66	0·00	2·28	2·54	0·20	0·31	9·66
		More than half the week (with interrupted service)	Count	309	1282	3130	2	2240	842	1176	43	1211	451	3	15	10704
			%	56·18	93·37	78·51	0·19	41·99	58·51	54·60	10·70	17·14	39·56	0·15	0·51	36·37
		Constant supply	Count	146	1	516	1074	883	154	801	359	5672	465	470	2877	13418
			%	26·55	0·07	12·94	99·81	16·55	10·70	37·19	89·30	80·29	40·79	23·89	97·62	45·59
	Access to soap (n=29469 mothers)	No	Count	1	33	148	8	50	15	21	2	43	6	51	14	392
			%	0·18	2·40	3·71	0·74	0·94	1·04	0·97	0·50	0·61	0·53	2·57	0·48	1·33
		Yes	Count	549	1332	3845	1069	5228	1414	2132	399	6948	1106	1895	2928	28845
			%	99·81	96·94	96·29	99·26	97·92	98·19	98·89	99·50	98·27	96·93	95·66	99·36	97·88
		Sometimes	Count	0	9	0	0	61	11	3	0	79	29	35	5	232
			%	0·00	0·66	0·00	0·00	1·14	0·76	0·14	0·00	1·12	2·54	1·77	0·17	0·79
	Hand washing frequency (n=29471 mothers)	Occasionally	Count	163	1362	1889	1070	4637	1184	1533	184	3178	195	1612	556	17563
			%	29·64	99·13	47·30	99·34	86·84	82·22	71·09	45·77	44·95	17·09	81·37	18·87	59·59
		Frequently	Count	387	12	2105	7	702	256	623	218	3892	946	369	2391	11908
			%	70·36	0·87	52·70	0·65	13·15	17·78	28·90	54·23	55·05	82·91	18·63	81·13	40·41
		Occasionally	Count	112	1296	788	784	3441	724	482	119	1602	58	1481	75	10962

Domain	Characteristic	Categories		BC	BK	ES	IN	NK	NN	NW	PC	PP	RU	RK	ZAT	Total
	Bath or shower frequency (n=17563 mothers)		%	68·70	95·15	41·72	73·27	74·20	61·15	31·44	64·67	50·41	29·74	91·87	13·49	62·42
		Frequently	Count	51	66	1101	286	1196	460	1051	65	1576	137	131	481	6601
			%	31·29	4·85	58·28	26·73	25·79	38·85	68·56	35·33	49·59	70·26	8·13	86·51	37·58
Birth and neonatal	Gestational age (n=29017 neonates)	Term	Count	452	926	2313	883	4761	1203	2063	312	5485	912	1880	2032	23222
			%	80·28	69·62	82·58	79·41	85·31	78·58	92·97	76·09	76·49	77·75	93·86	65·04	80·03
		Pre-term	Count	72	338	399	217	157	291	72	57	1302	247	107	968	4227
			%	12·79	25·41	14·24	19·51	2·81	19·01	3·24	13·90	18·16	21·06	5·34	30·99	14·57
		Post-term	Count	39	66	89	12	662	37	84	41	384	14	16	124	1568
			%	6·93	4·96	3·18	1·08	11·86	2·42	3·79	10·00	5·35	1·19	0·80	3·97	5·40
	Premature rupture of membranes (n=29936 neonates)	No	Count	507	1333	3809	1053	4512	1334	2122	334	5738	1054	1888	2537	26221
			%	90·21	96·38	90·97	93·68	82·49	87·36	97·38	80·87	82·60	89·93	94·54	85·28	87·59
		Yes	Count	55	50	378	71	958	193	57	79	1209	118	109	438	3715
			%	9·78	3·62	9·03	6·32	17·51	12·64	2·62	19·13	17·40	10·07	5·46	14·72	12·41
	Delivered by C-section (n=30498 neonates)	No	Count	484	1094	2559	288	4509	958	1354	271	4700	624	1026	1439	19306
			%	85·97	78·93	61·96	25·58	80·75	62·57	60·88	65·30	65·32	53·20	51·17	45·45	63·30
		Yes	Count	79	292	1571	838	1075	573	870	144	2495	549	979	1727	11192
			%	14·03	21·07	38·04	74·42	19·25	37·43	39·12	34·70	34·68	46·80	48·83	54·55	36·70
	Emergency or planned C-section where recorded (some unknown) (n=11139)	Emergency	Count	50	289	1378	158	731	542	546	105	1848	493	947	968	8055
			%	63·29	99·66	88·22	18·85	68·64	94·59	62·83	73·94	74·76	89·95	97·03	56·12	72·31
		Planned	Count	29	1	184	680	334	31	323	37	624	55	29	757	3084
			%	36·71	0·34	11·78	81·15	31·36	5·41	37·17	26·06	25·24	10·04	2·97	43·88	27·69
	Assisted birth (n=30498 neonates)	No	Count	21	662	2119	269	4066	506	1083	153	2529	623	3	1354	13388
			%	3·73	47·76	51·31	23·89	72·82	33·05	48·70	36·87	35·15	53·11	0·15	42·77	43·90
		Yes	Count	542	724	2011	857	1518	1025	1141	262	4666	550	2002	1812	17110
			%	96·27	52·24	48·69	76·11	27·18	66·95	51·30	63·13	64·84	46·89	99·84	57·23	56·10
	Breech birth (n=30500 neonates)	No	Count	550	1355	3907	1119	5176	1377	2106	389	6890	1130	1973	2880	28852
			%	97·69	97·76	94·53	99·38	92·69	89·94	94·69	93·73	95·77	96·33	98·40	90·97	94·59
		Yes	Count	13	31	226	7	408	154	118	26	304	43	32	286	1648
			%	2·31	2·24	5·47	0·62	7·31	10·06	5·31	6·27	4·23	3·67	1·60	9·02	5·40
	Perinatal asphyxia (n=30550 neonates)	No	Count	491	1179	4048	1109	5315	1263	2144	395	6882	1082	1763	2274	27945
			%	87·20	85·06	96·80	98·49	95·18	82·50	96·40	95·18	95·65	92·24	87·93	71·83	91·47
		Yes	Count	70	207	134	16	235	264	75	19	282	90	241	807	2440
			%	12·43	14·94	3·20	1·42	4·21	17·24	3·37	4·58	3·92	7·67	12·02	25·49	7·99
		Don't know	Count	2	0	0	1	34	4	5	1	31	1	1	85	165
			%	0·36	0·00	0·00	0·09	0·61	0·26	0·22	0·24	0·43	0·09	0·05	2·68	0·54
	Is current birth a multiple? (n=30557 neonates)	No	Count	537	1362	3826	1028	5102	1366	2085	389	6959	1117	1957	2737	28465
			%	95·38	98·27	91·38	91·30	91·37	89·22	93·75	93·73	96·69	95·23	97·61	86·45	93·15

Domain	Characteristic	Categories		BC	BK	ES	IN	NK	NN	NW	PC	PP	RU	RK	ZAT	Total
		Yes	Count	26	24	361	98	482	165	139	26	238	56	48	429	2092
			%	4·62	1·73	8·62	8·70	8·63	10·78	6·25	6·27	3·31	4·77	2·39	13·55	6·85

**Descriptive statistics are based on available cases.*

Supplementary Table 9: All-cause mortality incidence rates in facility-based births across seven low-income and middle-income countries

	No sepsis				Clinically-suspected sepsis but not laboratory-confirmed				Clinically-suspected and laboratory-confirmed sepsis				Total			
**Site**	Number of neonates included in analysis	Number deceased	Number of observed days	Incidence of mortality per 1000 neonate-days*	Number of neonates included in analysis	Number deceased	Number of observed days	Incidence of mortality per 1000 neonate-days*	Number of neonates included in analysis	Number deceased	Number of observed days	Incidence of mortality per 1000 neonate-days*	Number of neonates included in analysis	Number deceased	Number of observed days	Incidence of mortality per 1000 neonate-days*
**BC-Bangladesh**	437	16	18586	0·86 (0·49 to 1·40)	89	2	4689	0·43 (0·05 to 1·54)	37	4	1696	2·36 (0·64 to 6·04)	563	22	24970	0·88 (0·55 to 1·33)
**BK-Bangladesh**	1351	29	41909	0·69 (0·46 to 0·99)	31	3	619	4·85 (1·00 to 14·16)	4	1	64	15·63 (0·40 to 87·06)	1386	33	42591	0·77 (0·53 to 1·09)
**ES-Ethiopia**	3708	53	125456	0·42 (0·32 to 0·55)	296	25	8573	2·92 (1·89 to 4·30)	183	24	5449	4·40 (2·82 to 6·55)	4187	102	139478	0·73 (0·60 to 0·89)
**IN-India**	1082	17	18330	0·93 (0·54 to 1·48)	36	3	944	3·18 (0·66 to 9·29)	8	2	345	5·80 (0·70 to 20·94)	1126	22	19619	1·12 (0·70 to 1·70)
**NK-Nigeria**	5053	65	267837	0·24 (0·19 to 0·31)	437	17	21947	0·77 (0·45 to 1·24)	94	4	4741	0·84 (0·23 to 2·16)	5584	86	294525	0·29 (0·23 to 0·36)
**NN-Nigeria**	1228	20	61927	0·32 (0·20 to 0·50)	202	38	5202	7·30 (5·17 to 10·03)	101	16	2783	5·75 (3·29 to 9·34)	1531	74	69911	1·06 (0·83 to 1·33)
**NW-Nigeria**	2122	25	105761	0·24 (0·15 to 0·35)	71	6	3025	1·98 (0·73 to 4·32)	31	3	1324	2·27 (0·47 to 6·62)	2224	34	110110	0·31 (0·21 to 0·43)
**PC-Pakistan**	314	7	8393	0·83 (0·34 to 1·72)	67	8	1142	7·01 (3·02 to 13·80)	34	11	607	18·12 (9·05 to 32·43)	415	26	10143	2·56 (1·67 to 3·76)
**PP-Pakistan**	4833	83	95290	0·87 (0·69 to 1·08)	1760	173	35239	4·91 (4·21 to 5·70)	604	92	9172	10·03 (8·09 to 12·30)	7197	348	139702	2·49 (2·24 to 2·77)
**RU-Rwanda**	874	10	2441	4·10 (1·96 to 7·53)	249	26	4461	5·83 (3·81 to 8·54)	50	5	903	5·54 (1·80 to 12·92)	1173	41	7805	5·25 (3·77 to 7·13)
**RK-Rwanda**	1524	3	12036	0·25 (0·05 to 0·73)	332	10	3111	3·21 (1·54 to 5·91)	149	6	1385	4·33 (1·59 to 9·43)	2005	19	16532	1·15 (0·69 to 1·79)
**ZAT-South Africa**	2606	35	149381	0·23 (0·16 to 0·33)	483	20	26600	0·75 (0·46 to 1·16)	77	15	3916	3·83 (2·14 to 6·32)	3166	70	179898	0·39 (0·30 to 0·49)
**Total**	25132	363	907348	0·40 (0·25 to 0·62)	4053	331	115552	2·86 (1·34 to 7·60)	1372	183	32384	5·65 (3·00 to 13·35)	30557	877	1055284	0.83 (0.37 to 2.00)

**Values in parentheses are 95% confidence intervals inflated for clustering of neonates within sites.*

Supplementary Data 1: Associations with clinically suspected sepsis – Bivariable results

A higher risk of clinically suspected sepsis was associated with the following health care factors; being situated on a neonatal or obstetrics and gynaecology ward compared to a maternity ward (Neonatal: RR: 7·46 95% CI: 6·66-7·91) (O&G: RR: 1·13 95% CI: 1·03-1·25), a bathroom on the ward (RR: 1·56 95% CI: 1·36-1·79), being located nearest to the ward entrance (RR: 1·12 95% CI: 1·04-1·20) or being located in the middle of the ward (RR: 1·22 95% CI: 1·14-1·31) compared to being furthest away from the ward entrance door. Another factor which was associated with higher risk of clinically suspected sepsis was when the ward had more than three beds. (Appendix P33-36).

Statistically significant associations with a higher risk of clinically suspected sepsis linked to maternal factors include maternal use of antibiotics in the three months prior to enrolment (RR: 1·53 95% CI: 1·41-1·66), being hospitalised in the prior 12 months (RR: 1·40 95% CI: 1·29 to 1·52). Maternal health conditions associated with a higher risk of clinically suspected sepsis included diabetes (RR: 1·27 95% CI: 1·12-1·45), hypertension (RR: 1·42 95% CI: 1·32-1·53), immune-compromise (RR: 1·59 95% CI: 1·44-1·76), malaria (RR: 1·30 95% CI: 1·17-1·44), and infection (RR: 1·67 95% CI: 1·47-1·90). Mothers who accessed private or traditional healthcare in the three months prior to enrolment (PH: RR: 1·19 95% CI: 1·12-1·27 or TH: RR: 1·18 95% CI: 1·06-1·31) Mothers in their first pregnancy were also found to have a higher risk of clinically suspected sepsis, compared to those who have had previous livebirth. (Appendix P33-36).

Birth and neonatal factors associated with higher risk of clinically suspected sepsis included pre-term delivery (RR: 3·50 95% CI: 3·33-3·66), premature rupture of membranes (RR: 1·91 95% CI: 1·81-2·01), Caesarean section delivery (elective or emergency) compared to spontaneous vaginal delivery (Elective: RR: 1·51 95% CI: 1·38-1·65, Emergency: RR: 2·26 95% CI: 2·13-2·39), perinatal asphyxia (RR: 3·32 95% CI: 3·15-3·50), breech delivery (RR: 1·90 95% CI: 1·77-2·04) and being part of a multiple birth (RR: 1·36 95% CI: 1·24-1·49) were also associated with higher risk of clinically suspected sepsis (Appendix P33-36).

Living environment factors associated with a higher risk of clinically suspected sepsis included having a higher than average household income (RR: 1·18 95% CI: 1·09-1·27), primary source of drinking water being groundwater, private well or communal taps compared to municipal network (GW: RR: 1·37 95% CI: 1·21-1·54) (PW: RR: 1·13 95% CI: 1·06-1·21) (CT:RR: 1·21

95% CI: 1·11-1·31) and households served by a wastewater network (RR: 1·24 95% CI: 1·18-1·32). Other factors included living in a rural area (RR: 1·27 95% CI: 1·21-1·34), the presence of stagnant water or sewage near to the home (RR: 1·08 95% CI: 1·00-1·17). Finally, showering occasionally as opposed to frequently and having no bedrooms in the home compared to one or more bedrooms were factors also associated higher risk of with clinically suspected sepsis (Appendix P33-36).

Supplementary Table 10: Bivariable results for associations with clinically suspected sepsis*

Domain	Variable	Categories	RR	95% CI
Healthcare	Type of ward	Maternity	Reference Category	
		Neonatal	7·26	6·66 to 7·91
		Obstetrics & Gynaecology	1·13	1·03 to 1·25
		Others	0·82	0·68 to 1·00
	Number of beds on the ward	1-3	Reference Category	
		4-10	3·15	2·46 to 4·02
		11-20	7·48	5·77 to 9·69
		21+	4·13	3·01 to 5·67
	Bathroom on the ward	Yes	1·56	1·36 to 1·79
	Mothers' location on the ward	First 1/3 (closest to door)	1·12	1·04 to 1·20
		Middle 1/3	1·22	1·14 to 1·31
		Last 1/3 (furthest from door)	Reference Category	
		Other	1·46	1·24 to 1·73
Maternal	Mothers age in years	Up to 20	Reference Category	
		21 to 35	0·99	0·92 to 1·08
		36 and above	1·08	0·97 to 1·19
	Pregnancy history	First pregnancy	Reference Category	
		Previous pregnancy didn't come to term	1·06	1·00 to 1·12
		Live previous birth	0·82	0·77 to 0·86
	Health conditions	Diabetes	1·27	1·12 to 1·45
		Hypertension	1·42	1·32 to 1·53
		Immune compromised	1·59	1·44 to 1·76
		Malaria	1·30	1·17 to 1·44
		TB	0·91	0·48 to 1·70
		Infection	1·67	1·47 to 1·90
		Typhoid	1·00	0·68 to 1·48

		Other health condition	0·64	0·60 to 0·67
	Received private healthcare in the previous 3 months	Yes	1·19	1·12 to 1·27
	Visited traditional healer in the previous 3 months	Yes	1·18	1·06 to 1·31
	Hospitalised in the previous 12 months	Yes	1·40	1·29 to 1·52
	Used antibiotics in the previous 3 months	Yes	1·53	1·41 to 1·66
	Mothers' educational status	None or Limited	Reference Category	
		Secondary schooling or University	0·96	0·91 to 1·02
Living environment	Member of household travelled outside city, province, or country in previous 12 months	Yes	1·04	0·98 to 1·10
	Household income	Household income is below average	Reference Category	
		Household income is equal or above average	1·18	1·09 to 1·27
	Type of area	Rural	1·27	1·20 to 1·34
		Urban	Reference Category	
		Semi-rural	1·04	0·93 to 1·15
	Type of house	Apartment	Reference Category	
		Separate house	1·10	1·03 to 1·18
		Shack	1·02	0·91 to 1·15
		Other	1·25	0·95 to 1·64
	Number of bedrooms in residence	0	Reference Category	
		1-2	0·75	0·63 to 0·87
		3+	0·68	0·57 to 0·80
	Number of people residing in the house	1-3	Reference Category	
		4-6	0·87	0·82 to 0·92
		7+	0·94	0·88 to 1·00

	Primary source of drinking water	Municipal network	Reference Category	
		Private well	1·13	1·06 to 1·21
		Communal taps	1·21	1·11 to 1·31
		Water vender, sachet or bottled water	1·06	0·95 to 1·18
		Ground water	1·37	1·21 to 1·54
	Is the water treated	Boiled	Reference Category	
		Filtered	0·93	0·83 to 1·04
		Neither	0·96	0·88 to 1·05
	Presence of stagnant or sewage water near home	Yes	1·08	1·00 to 1·17
	Electricity supply in household	No supply	Reference Category	
		Poor supply	0·61	0·53 to 0·69
		Intermittent supply	0·53	0·47 to 0·59
		Regular supply	0·67	0·60 to 0·74
	Frequency of solid waste collection, if there is a pipe near	No solid waste nearby or "other"	Reference Category	
		We deal with it ourselves	0·95	0·86 to 1·05
		Once a week or more	1·05	0·97 to 1·13
		Less than once a week	0·70	0·58 to 0·85
	Type of toilet	Pit latrine or No toilet	Reference Category	
		Sit and or squat with flush	0·84	0·77 to 0·93
	Whether house is served by wastewater network	Yes	1·24	1·18 to 1·32
	Access to soap (of mother)	Yes	1·13	0·89 to 1·45
	Hand washing frequency (of mother)	Occasionally	Reference Category	
		Frequently	0·95	0·91 to 1·00
	Bath or shower frequency (of mother)	Occasionally	Reference Category	
		Frequently	0·92	0·86 to 0.99

Birth and neonatal	Gestational age	Term	Reference Category	
		Preterm	3·50	3·33 to 3·66
		Postterm	1·06	0·93 to 1·21
	Premature rupture of membranes	Yes	1·91	1·81 to 2·01
	Delivery type	Natural birth	Reference Category	
		Planned C-section	1·51	1·38 to 1·65
		Emergency C-section	2·26	2·13 to 2·39
		Other assisted birth	0·93	0·86 to 1.01
	Breech birth	Yes	1·90	1·77 to 2·04
	Perinatal asphyxia	No	Reference Category	
		Yes	3·32	3·15 to 3·50
		Don't know	1·80	1·37 to 2·36
	Multiple birth	Yes	1·36	1·24 to 1·49

**Models based on multiply imputed data (n=30557).*

Supplementary Table 11: Bivariable results for associations with LCS*

Domain	Variable	Categories	RR	95% CI
Healthcare	Type of ward	Maternity	Reference Category	
		Neonatal	7·73	6·31 to 9·46
		Obstetrics & Gynaecology	1·08	0·87 to 1·34
		Others	0·69	0·48 to 0·99
	Number of beds on the ward	1-3	Reference Category	
		4-10	1·87	1·17 to 2·99
		11-20	5·28	3·20 to 8·73
		21+	2·24	1·13 to 4·44
	Bathroom on the ward	Yes	1·78	1·28 to 2·46
	Mother's location on the ward	First 1/3 (closest to door)	1·42	1·21 to 1·67
		Middle 1/3	1·42	1·20 to 1·67
		Last 1/3 (furthest from door)	Reference Category	
		Other	1·74	1·18 to 2·56
Maternal	Mothers age in years	Up to 20	Reference Category	
		21 to 35	0·89	0·76 to 1·06
		36 and above	1·01	0·81 to 1·27
	Pregnancy history	First pregnancy	Reference Category	
		Previous pregnancy didn't come to term	1·02	0·90 to 1·16
		Live previous birth	0·83	0·74 to 0·94
	Health conditions	Diabetes	1·39	1·02 to 1·90
		Hypertension	1·73	1·47 to 2·04
		Immune compromised	1·52	1·19 to 1·93
		Malaria	1·35	1·08 to 1·70
		TB	0·66	0·10 to 4·39
		Infection	1·87	1·39 to 2·51
		Typhoid	0·57	0·19 to 1·76

		Other health condition	0·59	0·52 to 0·66
	Received private healthcare in the previous 3 months	Yes	1·09	0·95 to 1·25
	Visited traditional healer in the previous 3 months	Yes	1·36	1·07 to 1·72
	Hospitalised in the previous 12 months	Yes	1·53	1·27 to 1·85
	Used antibiotics in the previous 3 months	Yes	1·48	1·22 to 1·82
	Mother's educational status	None or Limited	Reference Category	
		Secondary schooling or University	1·05	0·93 to 1·19
Living environment	Member of household travelled outside city, province, or country in previous 12 months	Yes	0·98	0·86 to 1·12
	Household income	Household income is below average	Reference Category	
		Household income is equal or above average	1·22	1·03 to 1·44
	Type of area	Rural	1·26	1·11 to 1·43
		Urban	Reference Category	
		Semi-rural	1·09	0.88 to 1.36
	Type of house	Apartment	Reference Category	
		Separate house	1·06	0·91 to 1·24
		Shack	1·19	0·90 to 1·57
		Other	1·17	0·69 to 1·97
	Number of bedrooms in residence	0	Reference Category	
		1-2	0·90	0·67 to 1·22
		3+	0·80	0·58 to 1·10
	Number of people residing in the house	2-3	Reference Category	
		4-6	0·84	0·74 to 0·95
		7+	0·96	0·83 to 1·11

	Primary source of drinking water	Municipal network	Reference Category	
		Private well	1·07	0·92 to 1·25
		Communal taps	1·15	0·96 to 1·38
		Water vender, sachet or bottled water	1·31	1·03 to 1·65
		Ground water	1·20	0·91 to 1·58
	Is the water treated	Boiled	Reference Category	
		Filtered	1·16	0·91 to 1·48
		Neither	1·07	0·89 to 1·29
	Presence of stagnant or sewage water near home	Yes	0·96	0·80 to 1·16
	Electricity supply in household	No supply	Reference Category	
		Poor supply	0·53	0·39 to 0·71
		Intermittent supply	0·51	0·41 to 0·64
		Regular supply	0·64	0·51 to 0·80
	Frequency of solid waste collection, if there is a pipe near	No solid waste nearby or "other"	Reference Category	
		We deal with it ourselves	1·03	0·84 to 1·27
		Once a week or more	1·09	0·93 to 1·30
		Less than once a week	0·75	0·51 to 1·10
	Type of toilet	Pit latrine or No toilet	Reference Category	
		Sit and or squat with flush	0·90	0·72 to 1·14
	Whether house is served by wastewater network	Yes	1·26	1·11 to 1·43
	Access to soap (of mother)	Yes	0·98	0·62 to 1·54
	Hand washing frequency (of mother)	Occasionally	Reference Category	
		Frequently	0·87	0·78 to 0·98
	Bath or shower frequency (of mother)	Occasionally	Reference Category	
		Frequently	0·94	0·80 to 1·10

Birth and neonatal	Pregnancy details	Term	Reference Category	
		Preterm	3·94	3·53 to 4·39
		Postterm	0·93	0·68 to 1·27
	Premature rupture of membranes	Yes	1·64	1·44 to 1·87
	Delivery type	Natural birth	Reference Category	
		Planned C-section	1·50	1·23 to 1·84
		Emergency C-section	1·92	1·69 to 2·18
		Other assisted birth	0·82	0·70 to 0·97
	Breech birth	Yes	2·02	1·70 to 2·41
	Perinatal asphyxia	No	Reference Category	
		Yes	3·82	3·38 to 4·31
		Don't know	2·43	1·36 to 4·33
	Multiple birth	Multiple birth	1·68	1·40 to 2·01

**Models based on multiply imputed data (n=30557).*

Supplementary Table 12: Differential association between premature rupture of membranes and LCS by timing of delivery

Variable	RR	Lower 95% CI	Upper 95% CI
Premature rupture of membranes and term birth	1·56	1·28	1·89
Premature rupture of membranes and preterm birth	0·99	0·83	1·19
Premature rupture of membranes and postterm birth	1·27	0·55	2·93

**Model is based on multiply imputed data (n=30557). Outcome is LCS. Models adjust for site and minimum sufficient adjustment set identified from DAGs (birth as part of a multiple, maternal hypertension, maternal age, maternal infection in the past three months, parity, type of residence, type of toilet in the home, primary source of drinking water, overall household income per month, and electricity supply in the home). P-value for interaction between gestational age and premature rupture of membrances = 0·004*

Supplementary Table 13: Bivariable results for associations with ACM*

Domain	Variable	Categories	HR	95% CI
Neonatal sepsis status	Sepsis status	No sepsis	Reference category	
		Clinically-suspected sepsis only	5·00	4·26 to 5·88
		Laboratory-confirmed sepsis	8·95	7·43 to 10·79
Healthcare	Type of ward	Maternity	Reference Category	
		Neonatal	1·88	1·38 to 2·55
		Obstetrics & Gynaecology	1·00	0·76 to 1·32
		Others	0·52	0·32 to 0·85
	Number of beds on the ward	1-3	Reference Category	
		4-10	1·55	0·79 to 3·01
		11-20	2·26	1·13 to 4·50
		21+	3·05	1·42 to 6·55
	Bathroom on the ward	Yes	0·89	0·63 to 1·26
	Mother's location on the ward	First 1/3 (closest to door)	0·96	0·80 to 1·16
		Middle 1/3	0·86	0·71 to 1·04
		Last 1/3 (furthest from door)	Reference Category	
		Other	1·47	0·96 to 2·25
Maternal	Mother's age	Up to 20	Reference Category	
		21 to 35	1·05	0·84 to 1·30
		36 and above	1·32	0·99 to 1·76
	Previous pregnancies	First pregnancy	Reference Category	
		Previous pregnancy didn't come to term	1·15	0·97 to 1·36
		Live previous birth	1·02	0·87 to 1·20
	Health conditions	Diabetes	1·06	0·72 to 1·56
		Hypertension	1·29	1·05 to 1·59
		Immune compromised	1·23	0·90 to 1·69
		Malaria	0·79	0·59 to 1·06
		TB	2·11	0·67 to 6·64
		Infection	1·24	0·86 to 1·78
		Typhoid	0·83	0·27 to 2·61
		Other health condition	0·87	0·74 to 1·03
	Healthcare contacts	Received private healthcare in the past 3 months	1·08	0·91 to 1·29
		Visited traditional healer in the past 3 months	1·28	0·97 to 1·68
		Hospitalised in the last 12 months	1·29	1·02 to 1·63
		Used antibiotics in the last 3 months	1·24	0.98 to 1·56
	Mother's education status	None or Limited	Reference Category	
		Secondary schooling or University	0·90	0·77 to 1·05

Domain	Variable	Categories	HR	95% CI
Living environment		Member of household travelled outside city, province, or country in previous 12 months	0·96	0·83 to 1·13
	Monthly household income	Household income is below average	Reference Category	
		Household income is equal or above average	1·27	1·01 to 1·60
	Type of area	Rural	1·20	1·02 to 1·41
		Urban	Reference Category	
		Semi-rural	0·90	0·67 to 1·20
	Type of residence	Apartment	Reference Category	
		Separate house	1·14	0·94 to 1·38
		Shack	1·37	1·00 to 1·87
		Other	1·23	0·59 to 2·56
	Number of bedrooms in residence	0	Reference Category	
		1-2	1·18	0·79 to 1·78
		3+	1·17	0·76 to 1·79
	Number of people in residence	1-3	Reference Category	
		4-6	0·89	0·76 to 1·04
		7+	0·82	0·68 to 1·00
	Primary source of drinking water	Municipal network	Reference Category	
		Private well	0·90	0·74 to 1·10
		Communal taps	0·89	0·70 to 1·14
		Water vender, sachet or bottled water	0·76	0·57 to 1·00
		Ground water	1·16	0·87 to 1·57
	Type of drinking water	Boiled	Reference Category	
		Filtered	0·83	0·58 to 1·19
		Neither	0·99	0·76 to 1·31
	Presence of stagnant or sewage water near home	Yes	1·00	0·81 to 1·23
	Solid waste pipe disposal	No solid waste nearby or “other”	Reference Category	
		We deal with it ourselves	1·20	0·94 to 1·54
		Once a week or more	1·09	0·88 to 1·35
		Less than once a week	0·90	0·57 to 1·43
	Type of toilet in residence	Pit latrine or no toilet	Reference Category	
		Sit and or squat with flush	0·86	0·65 to 1·12
		House is served by wastewater network	1·16	0·97 to 1·39
	Electricity in household	No supply	Reference Category	
		Poor supply	0·78	0·51 to 1·21
		Intermittent supply	0·89	0·62 to 1·27
		Regular supply	0·79	0·54 to 1·15
	Mother has access to soap	Yes	0·77	0·43 to 1·37
	Mother's handwashing frequency	Occasionally	Reference Category	

Domain	Variable	Categories	HR	95% CI
		Frequently	1·09	0·94 to 1·27
	Mother's shower or bath frequency	Occasionally	Reference Category	
		Frequently	1·02	0·85 to 1·23
Birth and neonatal	Gestational age	Term	Reference Category	
		Pre-term	4·00	3·41 to 4·69
		Post-term	0·73	0·45 to 1·18
		Premature rupture of membranes	1·09	0·92 to 1·29
	Delivery type	Natural birth	Reference Category	
		Planned C-section	0·81	0·63 to 1·04
		Emergency C-section	0·84	0·71 to 0·98
		Other assisted birth	0·55	0·44 to 0·69
		Breech birth	1·25	0·98 to 1·60
	Perinatal asphyxia	No	Reference Category	
		Yes	3·74	3·16 to 4·42
		Don't know	0·90	0·29 to 2·81
		Multiple birth	2·91	2·42 to 3·51

**Models based on multiply imputed data (n=30557).*

Supplementary Table 14: Statistically significant bivariable associations between healthcare, maternal, living environment, and birth and neonatal factors and clinically suspected sepsis, laboratory-confirmed sepsis and all-cause mortality*

			Clinically suspected sepsis		Laboratory confirmed sepsis		All-cause mortality	
**Domain**	**Variable**	**Categories**	**RR**	**95% CI**	**RR**	**95% CI**	**HR**	**95% CI**
Healthcare	Type of ward	Maternity	Reference Category					
		Neonatal	7·46	6·66 to 7·91	7·73	6·31 to 9·46	1·88	1·38 to 2·55
		Obstetrics and Gynaecology	1·13	1·03 to 1·25				
		Others	0·82	0·68 to 1·00	0·69	0·48 to 0·99	0·52	0·32 to 0·85
	Number of beds on the ward	1-3	Reference Category					
		4-10	3·15	2·46 to 4·02	1·87	1·17 to 2·99		
		11-20	7·48	5·77 to 9·69	5·28	3·20 to 8·73	2·26	1·13 to 4·50
		21+	4·13	3·01 to 5·67	2·24	1·13 to 4·44	3·05	1·42 to 6·55
	Bathroom on the ward	Yes	1·56	1·36 to 1·79	1·78	1·28 to 2·46		
	Mother's location on the ward	First 1/3 (closest to door)	1·12	1·04 to 1·20	1·42	1·21 to 1·67		
		Middle 1/3	1·22	1·14 to 1·31	1·42	1·21 to 1·67		
		Last 1/3 (furthest from door)	Reference Category					
		Other	1·56	1·32 to 1·83	1·87	1·30 to 2·71		
Maternal	Mother's age in years	Up to 20	Reference Category					
		21 to 35						
		36 and above						
	Pregnancy history	First pregnancy	Reference Category					
		Previous pregnancy did not result in a livebirth						
		Live previous birth	0·82	0·77 to 0·86	0·83	0·74 to 0·94		
	Health conditions	Diabetes	1·27	1·12 to 1·45	1·39	1·02 to 1·90		
		Hypertension	1·42	1·32 to 1·53	1·73	1·47 to 2·04	1·29	1·05 to 1·59
		Immune compromised	1·59	1·44 to 1·76	1·52	1·19 to 1·93		
		Malaria	1·30	1·17 to 1·44	1·35	1·08 to 1·70		
		TB						
		Infection	1·67	1·47 to 1·90	1·87	1·39 to 2·51		
		Typhoid						
		Other health condition	0·64	0·60 to 0·67	0·59	0·52 to 0·66		
	Received private healthcare in the previous 3 months	Yes	1·19	1·12 to 1·27				
	Visited traditional healer in the previous 3 months	Yes	1·18	1·06 to 1·31	1·36	1·07 to 1·72		
	Hospitalised in the previous 12 months	Yes	1·40	1·29 to 1·52	1·53	1·27 to 1·85	1·29	1·02 to 1·63
	Used antibiotics in the previous 3 months	Yes	1·53	1·41 to 1·66	1·48	1·22 to 1·82		
	Mother's educational status	None or Limited	Reference Category					
		Secondary schooling or University						
Living environment	Member of household travelled outside city, province, country in previous 12 months	Yes						

			Clinically suspected sepsis		Laboratory confirmed sepsis		All-cause mortality	
**Domain**	**Variable**	**Categories**	**RR**	**95% CI**	**RR**	**95% CI**	**HR**	**95% CI**
	Household income	Household income is below average	Reference Category					
		Household income is equal or above average	1·18	1·09 to 1·27	1·22	1·03 to 1·44	1·27	1·01 to 1·60
	Type of area	Rural	1.27	1.21 to 1.34	1·26	1·11 to 1·43	1·20	1·02 to 1·41
		Urban	Reference Category					
		Semi-rural						
	Type of house	Apartment	Reference Category					
		Separate house	1·10	1·03 to 1·18				
		Shack					1·37	1·00 to 1·87
		Other						
	Number of bedrooms in residence	0	Reference Category					
		1-2	0·75	0·63 to 0·87				
		3+	0·68	0·57 to 0·80				
	Number of people residing in the house	1-3	Reference Category					
		4-6	0·87	0·82 to 0·92	0·84	0·74 to 0·95		
		7+	0·94	0·88 to 1·00			0·82	0·68 to 1·00
	Primary source of drinking water	Municipal network	Reference Category					
		Private well	1·13	1·06 to 1·21				
		Communal taps	1·21	1·11 to 1·31				
		Water vender, sachet or bottled water			1·31	1·03 to 1·65	0·76	0·57 to 1·00
		Ground water	1·37	1·21 to 1·54				
	Is the water treated	Boiled	Reference Category					
		Filtered						
		Neither						
	Presence of stagnant or sewage water near home	Yes	1·08	1·00 to 1·17				
	Electricity supply in household	No supply	Reference Category					
		Poor supply	0·61	0·53 to 0·69	0·53	0·39 to 0·71		
		Intermittent supply	0·53	0·47 to 0·59	0·51	0·41 to 0·64		
		Regular supply	0·67	0·60 to 0·74	0·64	0·51 to 0·80		
	Frequency of solid waste collection, if there is a pipe near	No solid waste pipe nearby or "other"	Reference Category					
		We deal with it ourselves						
		Once a week or more						
	Type of toilet	Pit latrine or No toilet	Reference Category					
		Sit and or squat with flush	0·84	0·77 to 0·93				
	Whether house is served by wastewater network	Yes	1·24	1·18 to 1·32	1·26	1·11 to 1·43		
	Access to soap (of mother)	Yes						
	Hand washing frequency (of mother)	Occasionally	Reference Category					
		Frequently			0·87	0·78 to 0·98		

			Clinically suspected sepsis		Laboratory confirmed sepsis		All-cause mortality	
**Domain**	**Variable**	**Categories**	**RR**	**95% CI**	**RR**	**95% CI**	**HR**	**95% CI**
	Bath or shower frequency (of mother)	Occasionally	Reference Category					
		Frequently	0·92	0·86 to 0·99				
Birth and neonatal	Gestational age	Term	Reference Category					
		Pre-term	3·50	3·33 to 3·66	3·94	3·53 to 4·39	4·00	3·41 to 4·69
		Post-term						
	Premature rupture of membranes	Yes	1·91	1·81 to 2·01	1·64	1·44 to 1·87		
	Delivery type	Natural birth	Reference Category					
		Planned C-section	1·51	1·38 to 1·65	1·50	1·23 to 1·84		
		Emergency C-section	2·26	2·13 to 2·39	1·92	1·69 to 2·18	0·84	0·71 to 0·98
		Other assisted birth	0·92	0·85 to 0·99	0·82	0·70 to 0·97	0·55	0·44 to 0·69
	Breech birth	Yes	1·90	1·77 to 2·04	2·02	1·70 to 2·41		
	Perinatal asphyxia	No	Reference Category					
		Yes	3·32	3·15 to 3·50	3·82	3·38 to 4·31	3·74	3·16 to 4·42
		Don't know	1·80	1·37 to 2·36	2·43	1·36 to 4·33		
	Multiple birth	Yes	1·36	1·24 to 1·49	1·68	1·40 to 2·01	2·91	2·42 to 3·51

**Models based on multiply imputed data (n=30557).*

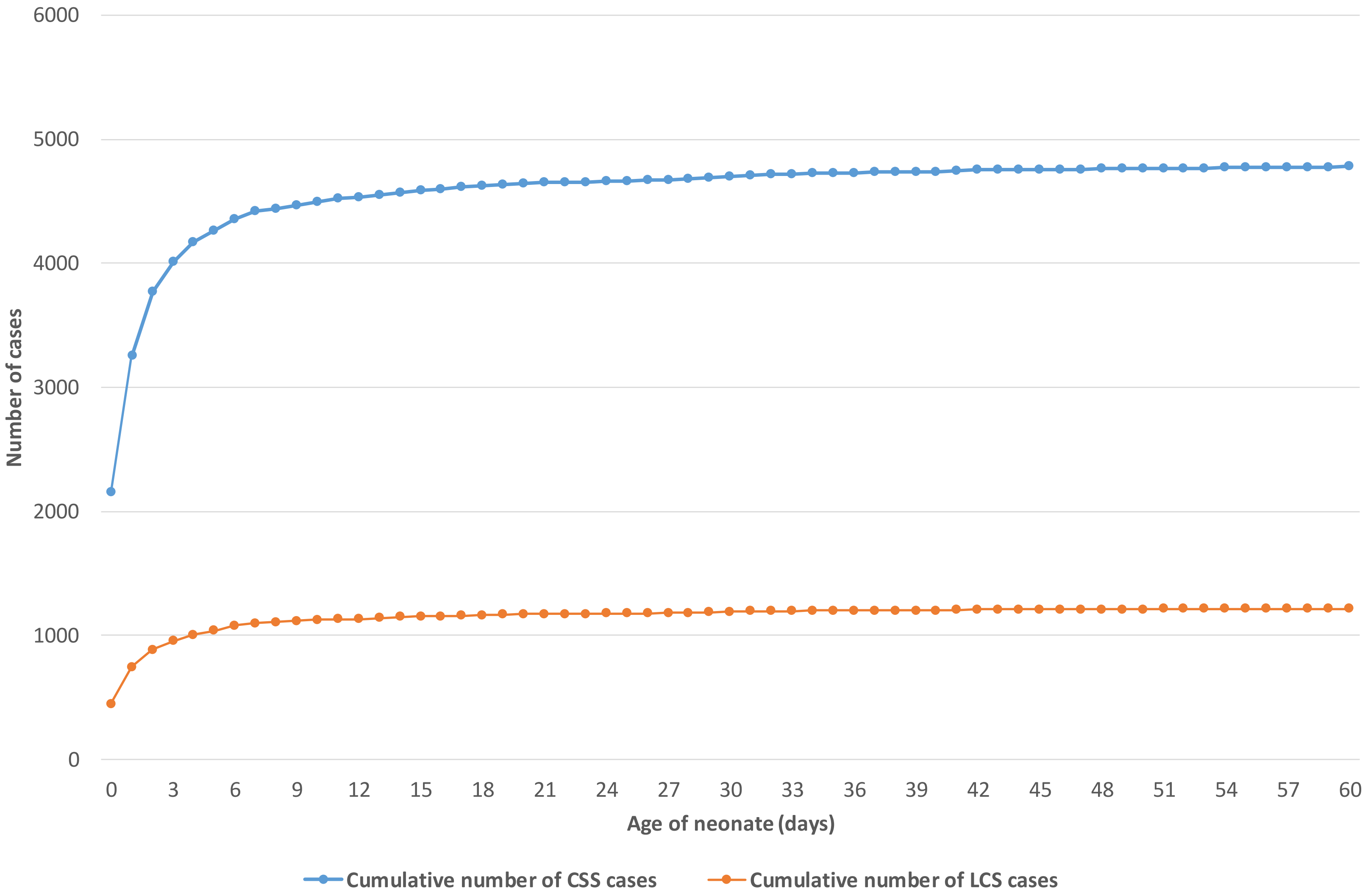

Supplementary Figure 6: Time to clinically suspected sepsis (CSS) and laboratory-confirmed sepsis (LCS). Cumulative number of CSS and LCS cases during the first 60 days of life in facility-born neonates in seven low-income and middle-income countries.

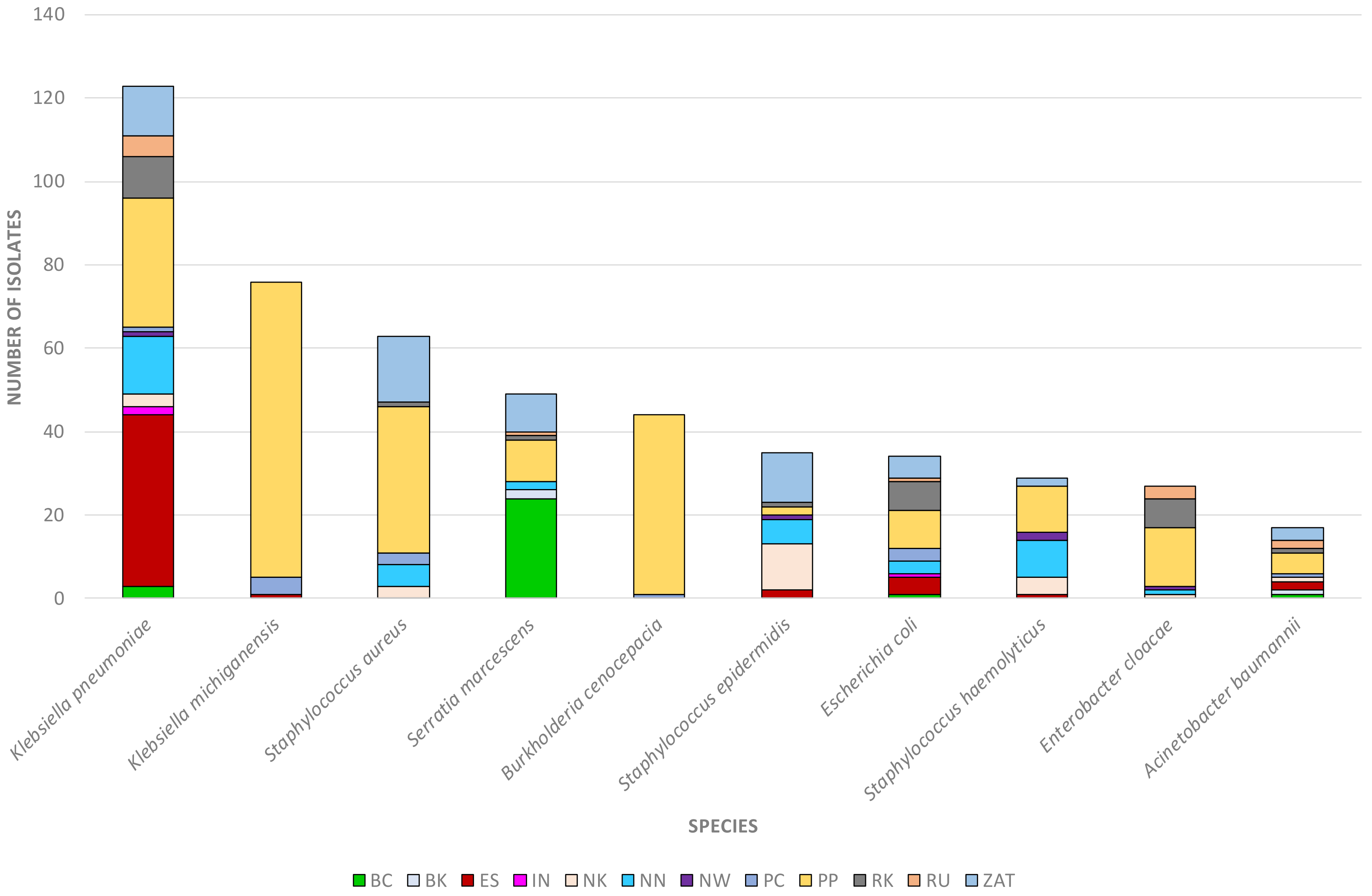

Supplementary Figure 7: Top ten species causing sepsis among facility-born neonates in seven low-income and middle-income countries
